# Tautomer aspects in the excited-state dynamics in 2-thiocytosine: intersystem crossing in the absence of the thiocarbonyl group†

**DOI:** 10.1039/d5sc01442e

**Published:** 2025-07-23

**Authors:** Bijay Duwal, Isabel Eder, Leticia González, Sebastian Mai, Susanne Ullrich

**Affiliations:** a Department of Physics and Astronomy, University of Georgia Athens Georgia 30602 USA ullrich@uga.edu; b Institute of Theoretical Chemistry, Faculty of Chemistry, University of Vienna Währinger Straße 17 1090 Vienna Austria sebastian.mai@univie.ac.at; c Vienna Research Platform on Accelerating Photoreaction Discovery, University of Vienna Währinger Straße 17 1090 Vienna Austria

## Abstract

Molecular tautomerism is ubiquitous in nature and plays a crucial role in regulating biological function. In nucleobases, for example, structural tautomerism not only influences base pairing and genetic coding in DNA but also modulates the molecular response to UV irradiation. The photostability of the nucleobases depends on efficient internal conversion and is highly sensitive to structural variations and micro-environmental effects. Among the pyrimidine bases, cytosine exhibits the greatest number of tautomeric forms, offering a rich landscape to explore diverse structural scenarios, while simultaneously posing significant experimental challenges. This study builds on that context by unveiling the gas-phase photophysics of 2-thiocytosine (2TC) from a unique tautomer perspective. Specifically, it elucidates the decay mechanism in the absence of a thiocarbonyl group but under the influence of chalcogen heavy atom substitution. In solution, 2TC exists in its thion form, whose photodynamics are characterized by efficient intersystem crossing to the triplet manifold. Electronic and structural factors associated with the thiocarbonyl group play a crucial role in suppressing internal conversion pathways to the ground state—pathways that are otherwise active in canonical cytosine. This ISC mechanism is tautomer specific and does not apply to the thiol form, which dominates in the gas phase. Time-resolved photoelectron spectroscopy of thiol-2TC reveals ultrafast internal conversion dynamics, alongside the emergence of a long-lived state with nanosecond lifetime. The latter distinguishes the photodynamics from its canonical counterpart, enol cytosine. *Ab initio* calculations provide detailed insights into the deactivation mechanism of thiol 2TC and clarify the differences on the effect of thionation on both tautomeric forms of cytosine. Finally, we discuss how protonation (and hydrogen bonding) can modulate intersystem crossing in thiobases, with broader implications to other thiocarbonyl-containing compounds.

## Abstract 2

Tautomerism of nucleobases and their thio-analogues plays a critical role in controlling their excited-state relaxation dynamics. We show how tautomerism—*i.e.*, the presence of a thiocarbonyl group—in 2-thiocytosine governs intersystem crossing.

## Graphical abstract

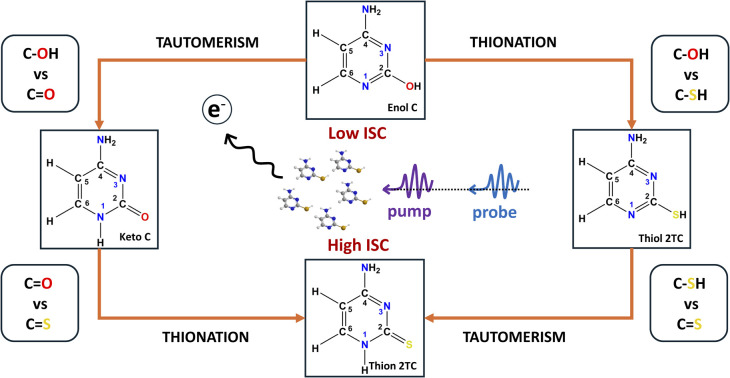

## Introduction

Tautomerism and single atom modifications of biomolecular building blocks have profound effects on a wide variety of biological functions. In the nucleic acids (DNA or RNA), base pairing and hydrogen bonding are highly sensitive to the location of protons in the nucleobases and tautomer interconversions can disrupt normal replication resulting in spontaneous mutations.^1^ Similarly, electronic and structural properties can be tuned through single atom editing, offering powerful means to control biochemical processes, as exemplified by photodynamic therapies in cancer treatments.^2–6^

**Fig. 1:**
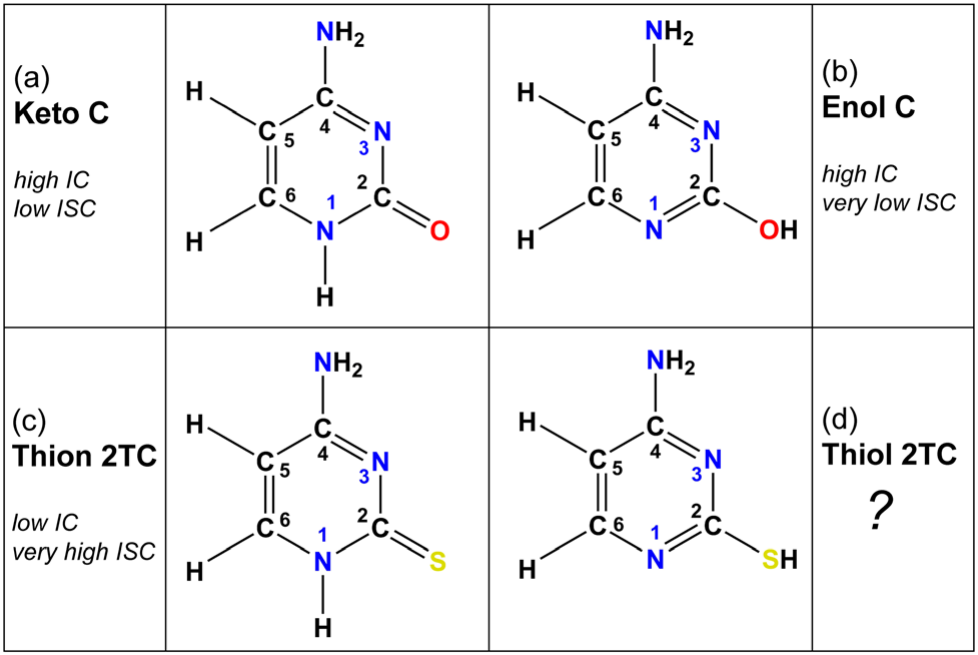
Molecular structures of the dominant cytosine (C) and 2-thiocytosine (2TC) tautomers. Both keto and enol tautomers of C are observable in the gas phase, whereas only the keto form is found in solution. 2TC exists purely in its thiol tautomeric form in the gas phase, but in the solution phase, its thion tautomeric form is strongly favored. “High IC” refers to the presence of easily accessible conical intersections between the excited ^1^nπ*/^1^ππ* states and the ground state and hence efficient internal conversion, whereas “low IC” denotes inefficient access to such a conical intersection. “Very high ISC” indicates near-unity triplet yield after intersystem crossing from the dark ^1^nπ* state, whereas “low/very low ISC” refers to minor triplet population after the decay from the dark ^1^nπ* state.

Upon UV photoexcitation, the nucleobases undergo ultrafast non-radiative relaxation. Conical intersections (CIs) between ground and excited state potential energy surfaces play a prominent role in facilitating internal conversion (IC) back to the ground state, while intersystem crossing (ISC) into the triplet manifold is also significant in several nucleobases.^7–11^ Beyond these general similarities, each tautomer may exhibit a distinct deactivation mechanism, as their excited state landscapes are highly sensitive to structural and environmental factors. These effects are intriguing, and investigating tautomer-dependent photodynamics experimentally remains challenging. While femtosecond pump–probe spectroscopies can monitor ultrafast deactivation dynamics, they lack the spectral resolution needed to distinguish between tautomers. Conversely, picosecond and nanosecond experiments offer tautomer selectivity but are unable to time-resolve the ultrafast processes and might even fail to detect tautomers with short-lived excited states. Despite these challenges, several studies have dedicated efforts to characterize tautomer-specific deactivation mechanisms. For example, among the purine bases, guanine shows the richest tautomerism, primarily the four tautomers: 9*H*- and 7*H*-amino keto and 9*H*- and 7*H*-amino enol while imino tautomers play a lesser role. Both 9*H*- and 7*H*-amino keto tautomers of guanine can internally convert from the lowest excited state back to the ground state *via* a C_2_ puckered CI. For 9*H*- and 7*H*-amino keto tautomers, access to the CI is barrierless and their lifetimes are therefore ultrashort. In the case of 9*H*- and 7*H*-amino enol tautomers, a barrier along the path slows down the dynamics.^12–15^ Similarly, the two 7*H*-amino and 9*H*-amino tautomers of adenine show different photophysical behavior. In both tautomers, C_2_-puckering and C_6_-puckering connect the lowest excited state to a CI with the ground state, however access barriers are such, that the 7*H* tautomer favors the C_6_ path whereas the 9*H* tautomer uses the C_2_ path and also deactivates faster.^14^ Among the pyrimidine nucleobases, thymine and uracil mostly exist in their diketo form,^16^ whereas cytosine exhibits complex tautomer specific photodynamics. Cytosine, found in both DNA and RNA, exists in various keto and enol forms in the gas phase.^17,18^ The structures of the two dominant tautomers of cytosine are displayed in Fig. 1a and b. Insights into the photodynamics of these molecules are typically obtained through a combination of experiment and theory, with the latter providing mechanistic details that describe experimental observations. Following photoexcitation, both tautomers of cytosine undergo complex deactivation dynamics along a number of competing pathways. *Ab initio* calculations and dynamics simulations have helped disentangling different pathways observed in experimental data,^19,20^ providing a good understanding of the contributions of individual tautomers.^17,18,21–23^ In the keto form of cytosine, the main pathways were identified as IC from the bright ^1^ππ* state either directly back to the ground state or to a dark intermediate of ^1^nπ* character. The latter subsequently undergoes ISC to the triplet manifold or internally converts back to the ground state. In contrast, for the enol form, the predominant pathways involve a two-step IC process from the bright ^1^ππ* state back to the ground state *via* the dark ^1^nπ* state, with very low propensity to form triplet states.

Just as tautomerism affects the nucleobases' behavior, their photodynamical properties are also extremely sensitive to other single-atom structural modifications. In thiobases, the replacement of an oxygen atom by sulfur induces characteristic changes in the topography of the excited-state potential energy surfaces.^6,7,24^ Upon substitution by this heavier chalcogen atom, the valence shell expands and shifts farther from the nucleus, leading to a lowering of the excitation energies of the singlet excited states that originate from sulfur localized orbitals.^25^ This effect is observed as a significant redshift of the UV absorption spectrum from the UVC region for the canonical nucleobases to the UVA region for thiocarbonyl-containing thiobases. According to *ab initio* calculations, a localized stabilization occurs in parts of the potential energy surfaces surrounding the excited state minima and is most pronounced for transitions with a high degree of sulfur localization, *e.g.*, the ^1^n_S_π* state. Because the CIs in thiobases do not stabilize to the same extent and become inaccessible from the deep minima, the molecule is trapped in the excited state. Additionally, the substitution of oxygen by a heavier chalcogen enhances the singlet-triplet spin–orbit couplings (SOCs). As a result, thiobases containing a thiocarbonyl group, exhibit altered excited state topographies that block IC pathways to the ground state and significantly enhance the rate of ISC to the triplet manifold—sometimes with near-unity yields. This behavior has been confirmed as a general characteristic by numerous studies combining nonadiabatic dynamics simulation with transient absorption spectroscopy in solution,^6–8,24–27^ as well as time-resolved photoelectron experiments in the gas phase.^27–32^

Guanine, adenine, and cytosine serve as ideal systems to gain insight into tautomerism and the effect of single atom substitution. In fact, the effect of thionation on the keto and enol tautomers of guanine have been recently investigated.^33,34^ Based on *ab initio* calculation,^33^ thion 6-thioguanine rapidly internally converts onto the lowest singlet state, ^1^nπ*, and from there branches to crossing points either back to the ground state or into the triplet manifold. Compared to keto guanine, the latter ISC pathway is strongly enhanced by the presence of the thiocarbonyl group which increases the SOC. In principle, in thiol 6-thioguanine the same IC and ISC pathways are available to repopulate the ground state either directly or *via* the triplet manifold. *Ab initio* calculations predict that both tautomers of 6TG should be present in a gas-phase molecular beam environment, which poses a challenging scenario for experiments. Surprisingly, only the thiol 6TG tautomer was identified by double-resonance techniques and, due to limitation in the time-resolution of ps-to-ns REMPI spectroscopy, only the slower but likely minor ISC pathway was observed.^34^ In the solution phase,^35,36^ methylation of the thiocarbonyl/carbonyl group in guanine derivatives has been used to mimic the thiol/enol tautomer. Comparison to the unmethylated counterparts revealed significant alteration of the IC and ISC pathways in these molecules confirming tautomer-specific photodynamics. These previous studies set the stage for further, and more comprehensive, investigations into thionation effects on ISC in the presence of tautomerism.

2-Thiocytosine (2TC) is a thionated analog of the nucleobase cytosine. The photophysics of 2TC have received far less attention compared to other pyrimidine thiobases.^27–32,37–40^ This may be due to the predominance of thion (with a thio-carbonyl group) and thiol (with a thiol group) tautomeric forms of 2TC (shown in Fig. 1c and d) in solution and the gas phase, respectively. According to previous work^41^ that investigated solvatochromic effects on the UV-vis absorption spectrum of 2TC and its tautomer contributions, the thion form is expected to be the only tautomer in acetonitrile, dimethyl sulfoxide, ethanol, methanol, and water solutions. There is a possibility that the thiol form is present in small amounts in ethyl acetate solution. In contrast to the solution phase, the thiol form is the most stable and only tautomer in the gas phase, which has been verified by theory and matrix isolation experiments.^41–43^ Gas-phase experiments of 2TC therefore allow to exclusively observe the photodynamics of a thiol thiobase. As mentioned above, all other pyrimidine thiobases (thiouracils and thiothymines), except for 2TC, occur in the biologically relevant thion form in both gas and solution phases and follow a deactivation mechanism that is common to the thiocarbonyl bases.^24,44,45^ 2TC therefore presents a distinct case and provides a unique opportunity to study the effect of heavy chalcogen atom substitution on the photophysics of a thiobase from a different tautomeric perspective. Of particular interest is whether the photodynamics of thiol 2TC more closely resemble those of thion 2TC or enol cytosine—that is, whether the mere presence of the heavy sulfur atom is sufficient to enable efficient ISC, or if a thiocarbonyl group is required.

In the present work, time-resolved photoelectron spectroscopy (TRPES) is used to observe the decay dynamics of thiol 2TC in the gas phase. High level *ab initio* calculations of the deactivation pathways provide further insight into the underlying mechanistic details, with the goal of understanding electronic and structural factors that govern the tautomer-specific behavior. In thion thiobases, selective stabilization of excited state minima with sulfur localized transitions leads to excited state topographies that favor population trapping and funneling towards ISC. Furthermore, the thiocarbonyl group participates in an out-of-plane distortion to access the crossing point. By observing the dynamics from a different tautomer perspective, *i.e.*, in the presence of the thiol group, the role of the thiocarbonyl group can be assessed further. Specifically, it can be hypothesized that the thiol H-atom hinders the electron donating ability of the functional group and thereby limits the stabilization and hence trapping ability of the excited state minima. This raises the question of whether the thiol 2TC photodynamics rely on efficient IC, similar to enol cytosine, rather than on ISC as observed in thion 2TC.

## Methods

### Gas phase UV-vis absorption spectrum

The gas-phase absorption spectrum of 2TC is obtained with a custom-built UV-vis spectrometer. The setup consists of a deuterium lamp (L2D2, Hamamatsu Co.) that produces UV-visible light which is collimated by a plano-convex lens (focal length 40 mm) and propagates through a stainless-steel cell with a window-to-window pathlength of ∼350 mm. The transmitted light is focused by another plano-convex lens (focal length 30 mm) and coupled into a fiber optic spectrometer (HR4000, Ocean Optics, Inc.). Spectra are recorded employing the supplied OceanView (Ocean Optics, Inc.) software with typical integration times of 100 ms. Gas phase absorption spectra of the sample are recorded using the following procedure: the sample is inserted into the cell before it is vacuum sealed and pumped to rough vacuum. First, reference lamp spectrum and background spectrum are recorded through the cell. Then the cell is heated to ∼300 °C by means of a rope heater to evaporate the sample and the sample spectrum with identical integration time as the reference spectrum is recorded. The UV-vis absorption spectrum is obtained as the logarithm of the ratio of the reference spectrum to the sample spectrum. Even under these elevated temperatures, the presence of the thion tautomer is expected to be negligible. Assuming the previously reported 5.0 kcal mol^−1^ energy difference between the thiol and thion tautomers,^25^ the Boltzmann population ratio at 300 °C is 98.7% : 1.3%, respectively.

### TRPES

The details of the TRPES experiment are discussed elsewhere^46–48^ and therefore only a brief description is given here. A Ti:Sa oscillator (Mira 900, Coherent Inc.) seeds a regenerative amplifier (Legend Elite HE, Coherent Inc.), the latter of which pumps two optical parametric amplifiers (OPAs) with the ability to produce tunable UV pulses. The pulses from the first OPA (OPerA, Coherent Inc.), which act as photoexciting pumps for the 2TC (Sigma-Aldrich, 97%) sample, are set to 243 nm, 250 nm, 260 nm, 270 nm, 280 nm, 290 nm, and 300 nm with a pulse energy of 2.0 μJ per pulse. The output of another OPA (TOPAS-C, Light Conversion Inc.), is set to 330 nm with a pulse energy of about 4.0 μJ and serves as a two-photon ionizing probe. The pump intersects the time-delayed probe in the ionization region of a magnetic bottle photoelectron spectrometer. The sample powder is evaporated from a heated (∼210 °C) quartz sample holder inside a pinhole nozzle and transported by a helium carrier gas through two skimmers and into the ionization region of the spectrometer. Assuming that the tautomer population ratio from the source is frozen out in the expansion, a molecular beam with 99.5% thiol 2TC is generated.^25^ TRPES are obtained with the magnetic bottle photoelectron spectrometer which makes time-of-flight (TOF) measurements of photoelectrons at different pump–probe delays. The probe delay is introduced with a self-aligning optical delay line (Smart Delay Line, Ultrafast Systems). The ultrafast dynamics are captured with an equal step size of 25 fs from −1 to 4 ps and the remaining range with unequal, gradually increasing steps up to a maximum delay of 3 ns. Measurements of 1,3-butadiene are used for timing calibrations and for conversion from TOF to photoelectron kinetic energies. The instrument response function exhibits a Gaussian full width at half maximum between 170 fs to 180 fs, depending on the pump wavelengths. Global lifetime analysis of the TRPES spectra was performed in Glotaran^49^ and Origin^50^ was employed for all other data analysis.

### Theoretical details

All relevant critical geometries (2 S_0_, 3 S_1_, 3 T_1_ minima, 9 S_1_/S_0_ and 2 T_2_/T_1_ CIs, 2 S_1_/T_2_ and 9 T_1_/S_0_ minimum-energy crossings, including rotamers, for 30 structures in total) were optimized based on initial guesses from enol-cytosine^19^ and TDDFT-based preliminary surface hopping trajectories. We employed extended multistate complete active space second-order perturbation theory (XMS-CASPT2)^51^ as implemented in OpenMolcas v23.06 (ref. 52) with an active space of 10 electrons in 8 orbitals (6 π/π* orbitals from the pyrimidine ring and 2 nitrogen lone pairs, see Fig. S1†) and the ANO-R1 basis set.^53^ 10 singlets or 10 triplets were included in the state-averaging and XMS procedures. The IPEA shift^54^ was set to zero and an imaginary level shift^55^ of 0.2 a.u. was used. The optimizations were based on analytical XMS-CASPT2 gradients^51^ with Cholesky decomposition. All relevant paths (76 segments in total) between the 30 structures were interpolated using the IDPP method^56^ and single-point calculations with 10 singlets, 10 cationic doublets, and 10 triplets were performed, where oscillator strengths, spin–orbit couplings, and Dyson norms were computed. These interpolation scans serve the purpose of checking for active orbital consistency and for the presence of barriers, for which the scans provide upper bounds. All 30 optimized geometries are given in the ESI† in *XYZ* format. The theoretical investigations focus on critical geometries and interpolation scans to thoroughly map out IC and ISC pathways that are relevant to the interpretation of the experimental data. Simulating the dynamics at a sufficiently high level of theory, *i.e.* XMS-CASPT2, is computationally prohibitive due to the extended (few ns) time window of interest. Instead, exploratory nonadiabatic dynamics simulations (TDA-PBE/def2-SVP with D3BJ correction) using SHARC^57,58^ and ORCA^59^ were performed to capture all reaction pathways of potential relevance and to ensure that the decay from higher bright singlet states to the S_1_ (which is the starting point of the PES exploration) occurs on faster timescales than the time-resolution of the TRPES experiments.

## Results and discussion

### Absorption spectrum and relevant transitions

**Fig. 2:**
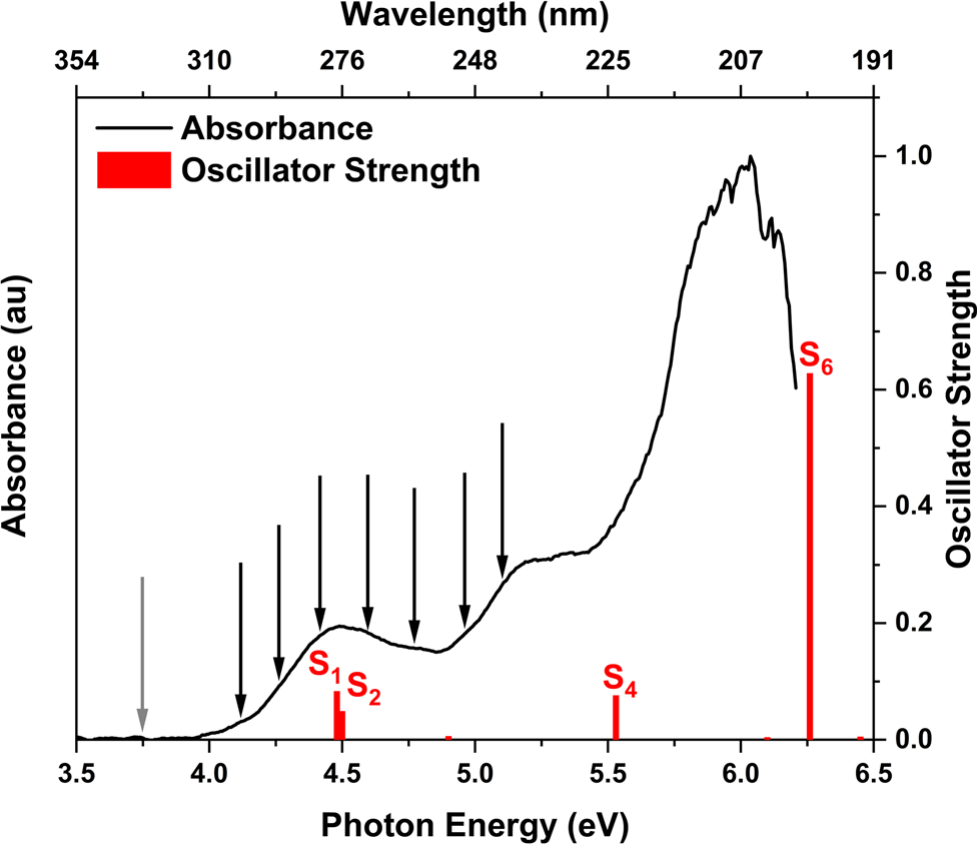
Experimental UV-vis absorption spectrum of 2TC in the gas phase. The vertical red lines indicate the computed vertical excitation energies of the singlet states of thiol 2TC with their heights denoting the oscillator strengths. The calculations are performed at the XMS-CASPT2(10,8)/ANO-R1 level of theory and the computed energy values are shifted by −0.3 eV to match the experimental absorption spectrum. The positions of the black arrows and grey arrow correspond to the photon energies of the pump and probe pulses, respectively.

The experimental gas phase UV-vis absorption spectrum of 2TC with superimposed theoretical transitions is shown in Fig. 2 and provides insight into the relevant photoexcited states of the thiol tautomer. The three main absorption bands are assigned to the lowest singlet excited states, S_1_/S_2_, S_4_, and S_6_ based on the results obtained from XMS-CASPT2(10,8)/ANO-R1 calculations. The computed vertical excitation energies and corresponding oscillator strengths of the singlet states of thiol 2TC are summarized in Table 1 and the adiabatic energies are provided in Table 2. When the theoretical values are red shifted by 0.3 eV, the adiabatic and vertical energies align well with the onset of the absorption spectrum and the three band maxima, respectively.

**Table 1:** Vertical excitation energies, oscillator strengths, and orbital characters of singlet and triplet states of thiol 2TC computed at the XMS-CASPT2(12,9)/ANO-R1 level of theory. See Table S1 for vertical excitation energies, oscillator strengths, and orbital characters of additional doublet and triplet states; see Fig. S1 for a depiction of the involved orbitals

States	Vertical excitation energy (eV)	Oscillator strength	Orbital characters
S_1_	4.78	0.083	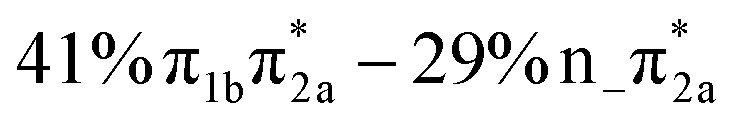
S_2_	4.80	0.049	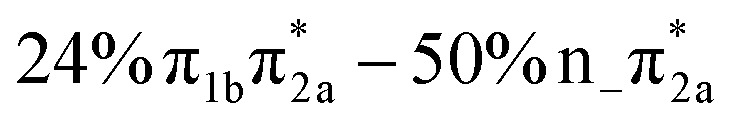
S_3_	5.20	0.006	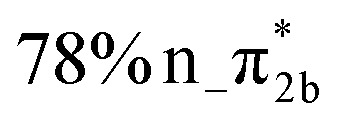
S_4_	5.83	0.076	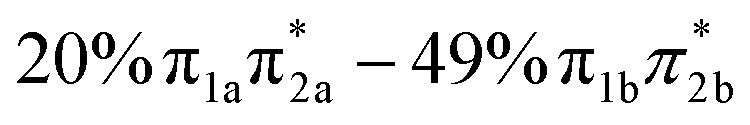
S_5_	6.40	0.004	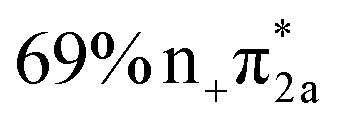
S_6_	6.56	0.628	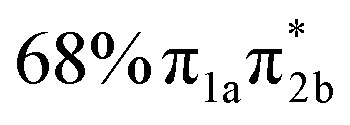
S_7_	6.75	0.005	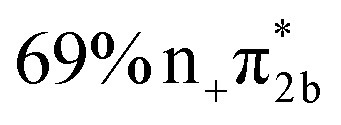
S_8_	7.55	0.108	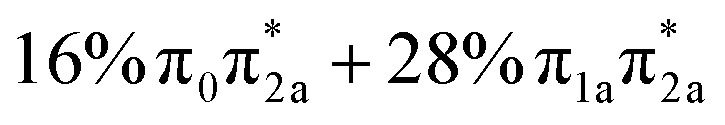
S_9_	8.24	0.040	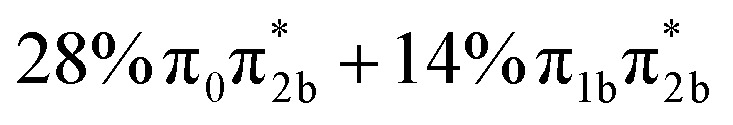
T_1_	4.25	—	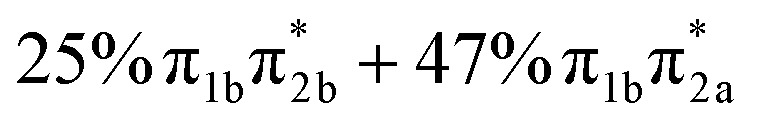
T_2_	4.55	—	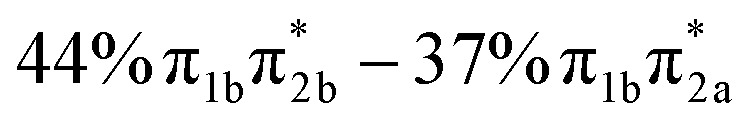
T_3_	4.63	—	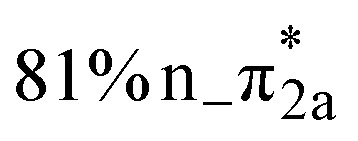

**Table 2:** Adiabatic excitation energies, adiabatic ionization energies, and ionization channels at relevant excited-state minima computed at XMS-CASPT2(12,9)/ANO-R1 level of theory. The adiabatic ionization energy represents the ionization potential at a specific geometry with respect to the energy of the S_0_ minimum

Geometry	Adiabatic excitation energy (eV)	Adiabatic ionization energy (eV)	Ionization channel (Dyson norm)
S_0_ min (b)	0.00	8.57	D_0_, π hole (0.87)
S_1_ min (nπ*)	4.13	9.42	D_0_, n hole (0.41)
S_1_ min (ππ* a)	4.39	9.80	D_0_, π hole (0.43)
S_1_ min (ππ* b)	4.41	9.51	D_0_, π hole (0.43)
T_1_ min (ππ* a)	3.57	9.47	D_0_, π hole (0.43)
T_1_ min (ππ* b)	3.58	9.50	D_0_, π hole (0.43)
T_1_ min (nπ*)	3.85	9.49	D_0_, n hole (0.39)

The main focus of the present study is to observe the decay dynamics of thiol 2TC photoexcited to its first bright state (
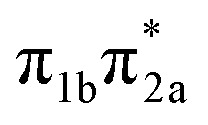
, corresponding to S_1_ or S_2_, see Table 1). The level of vibrational excitation of the first bright state increases at shorter pump wavelengths (black arrows in Fig. 2). At the shortest excitation wavelengths, the next higher bright state (
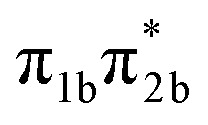
, S_4_ in Table 1) becomes potentially accessible. As a very fast deactivation from the higher excited states to the S_1_ can be assumed (see our discussion of corresponding dynamics simulations below), the theoretical analysis and potential energy scans focus only on the lowest singlet and triplet excited states as well as the ground state.

**Fig. 3:**
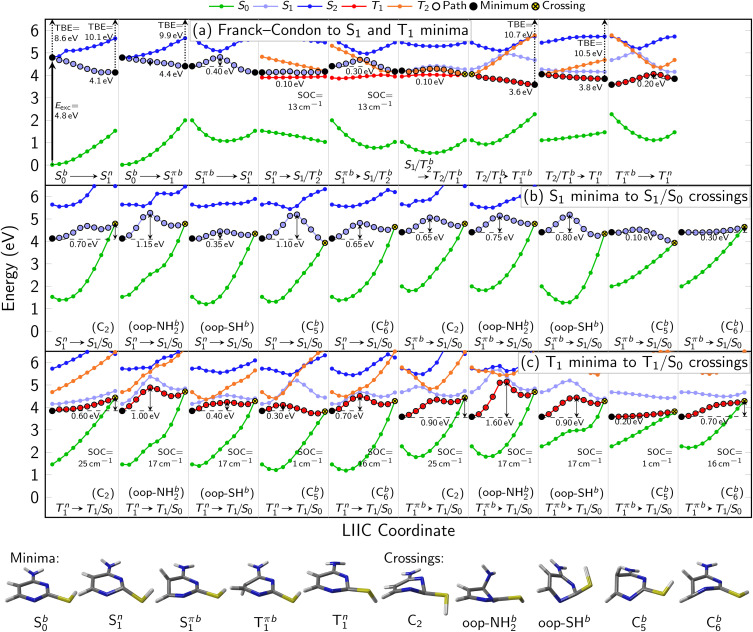
(a–c) Interpolation scans of potential energy surfaces connecting the most important critical points of the neutral states at the XMS-CASPT2(10,8)/ANO-R1 level of theory. The structures below panel (c) show the optimized minima of S_0_, S_1_ (^1^nπ*), S_1_ (^1^ππ*), and T_1_ as well as geometries representing the S_1_/S_0_ and T_1_/S_0_ crossing points. Vertical arrows indicate the computed excitation energy and total binding energies (TBE) at the minima. Due to the rather freely rotating SH group, most critical points have two rotamers (labeled as a and b). ESI Fig. S3–S6† show all PES interpolations for a larger number of states and for all rotamers, here we only show paths for rotamers b, which tend to exhibit smaller barriers. Table S4† lists important geometry parameters of all structures.

Table 2 collates the computed adiabatic excitation energies associated with relevant excited state minima, and adiabatic ionization energies (IE) of critical points along the theoretical relaxation paths. The latter is defined as the adiabatic excitation energy to a specific excited state minimum plus the vertical ionization energy from that minimum to the cationic ground state. Hence, it describes the changes in the ionization potential along a relaxation path. To aid the assignment of the TRPES spectra, additionally any changes in vibrational energy have to be taken into account. The vibrational excitation at a specific critical point is estimated as the difference between the vertical excitation energy (Table 1) and the adiabatic excitation energy (Table 2). In Fig. 3, total binding energy (TBE) is introduced, and it refers to the adiabatic ionization energy that also includes vibrational excitation. The TBE and orbital character are used for interpretation of the TRPES as discussed below and in the ESI (Fig. S2,† and Tables 2 and 3).

**Table 3:** Time constants *τ*_1_, *τ*_2_, and *τ*_3_ associated with a sequential decay model with three exponentials. For 300 nm, an additional Gaussian function was included in the fit to account for signals during the cross-correlation which may be due to probe–pump contributions. The errors are calculated based on the uncertainty in the reproducibility of results across multiple TRPES measurements at the same pump wavelength

Pump wavelength (nm)	Pump energy (eV)	*τ*_1_ (fs)	*τ*_2_ (ps)	*τ*_3_ (ns)
300	4.13	540 ± 40	13 ± 3	2.4 ± 0.6
290	4.28	300 ± 60	5.4 ± 0.4	3.6 ± 0.1
280	4.43	320 ± 30	3.5 ± 0.4	7 ± 1
270	4.60	230 ± 30	2.4 ± 0.1	8 ± 3
260	4.77	260 ± 30	2.0 ± 0.1	>8
250	4.96	120 ± 30	1.3 ± 0.1	>8
243	5.10	80 ± 30	1.2 ± 0.1	>8

### Overview over theoretical relaxation pathways

In Fig. 3, the most important segments of the excited-state potential energy surfaces (PESs) are depicted. Additional segments for the second rotamer and depictions of all structures are given in the ESI in Fig. S3–S6.† Fig. S3 and S4† also show the energies of the S_3_–S_5_ (and T_3_–T_5_) states, showing that any population of these higher states can immediately decay to the S_2_ and S_1_. This supports our focus on these low-lying singlet states in the following. At the Franck–Condon point, the lowest ^1^ππ* and ^1^nπ* states are essentially degenerate and mix considerably, leading to S_1_ and S_2_ being energetically very close and sharing the oscillator strength of the bright ^1^ππ* state. Following the adiabatic PES of the S_1_, three different S_1_ minima can be reached barrierlessly: an ^1^nπ* minimum at 4.1 eV and two ^1^ππ* minima (rotamers a and b) at 4.4 eV. Given that the initial (diabatic) electronic character will be predominantly ^1^ππ*, evolution toward the ^1^ππ* minima is expected to be favored. Once in the ^1^ππ* minima, the molecule can relax to the lower-lying ^1^nπ* minimum after crossing a 0.4 eV barrier. From any of the three S_1_ minima, the molecule can in principle return to the ground state or undergo ISC to the triplet state. Relaxation to the ground state is readily possible from the ^1^ππ* by overcoming a 0.1 eV barrier to reach a CI with strong C=C bond torsion (labeled “C_5_” in Fig. 3b). On the contrary, from the ^1^nπ* minimum, the relevant barriers are at least 0.35 eV (to reach the “out-of-plane SH” CI). ISC in contrast only requires overcoming a barrier of about 0.1 eV from the ^1^nπ* minimum. However, the small SOCs of only 13 cm^−1^ limit the ISC rate and yield. After ISC, the three T_1_ minima can easily be reached: a ^3^nπ* minimum at 3.8 eV and two ^3^ππ* rotamers at 3.6 eV. We note that the triplet ^3^ππ* minima are below the ^3^nπ* minimum, whereas the singlet ^1^ππ* minima are shifted above the ^1^nπ* minimum due to exchange repulsion that penalizes bright states.^60^ The ^3^nπ* minimum is separated by a barrier of 0.2 eV from the ^3^ππ* minima. Return to the ground state from the T_1_ minima can occur through different crossings, which either exhibit small barriers (0.2 eV) but negligible SOCs (1 cm^−1^) or slightly larger SOCs (17–25 cm^−1^) but larger barriers (at least 0.7 eV). Hence, we expect rather long-lived triplet states should they be populated. The T_1_/S_0_ crossings are closely related to the S_1_/S_0_ conical intersections.

We note that all the identified CIs and singlet-triplet crossings of thiol 2TC—including “oop-SH” and “oop-NH_2_”—are primarily characterized by some form of ring puckering. The “oop-SH” CI exhibits an out-of-plane deformation on the N_1_ and C_2_ atoms. The “C_2_” CI shows strong puckering on the C_2_ atom, the “oop-NH_2_” CI on atom N_3_, the “C_5_” CI on atom C_5_, and the “C_6_” CI on atoms C_6_ and N_1_ (Table S4† in SI). The “C_5_” and “C_6_” CIs are often referred to as “ethylene-like” CIs in the literature.^8,19,61–63^ As the out-of-plane vibrations of the different ring atoms are coupled to each other, we anticipate that vibrational energy can rather freely flow between the differing puckering modes and that the barrier height is the most important parameter that controls access to individual CIs. Nevertheless, access to the “oop-SH” and “oop-NH_2_” CIs might additionally be limited by the large-amplitude motion of the SH or NH_2_ groups.

In addition to the exploration of the potential energy surfaces in Fig. 3 and S3–S6,† we have also simulated the nonadiabatic dynamics of 2TC using surface hopping. As shown in Fig. S7,† depending on the initial conditions (molecule at 0 K or 500 K, excitation with 4.0–4.4 eV or 4.4–4.8 eV) the lifetime of the singlet states above the S_1_ is between 5 and 30 fs, well within the time resolution of our experiments. In the same time frame, the population of the triplet states remains essentially zero. This indicates that the higher singlet states directly deactivate to the S_1_ and do not further participate in the slower deactivation dynamics that is observed experimentally. Additionally, as shown in Fig. S8† the dynamics simulations indicate that under the employed conditions no dissociation of the SH group is to be expected.

### Time-resolved photoelectron spectra

**Fig. 4:**
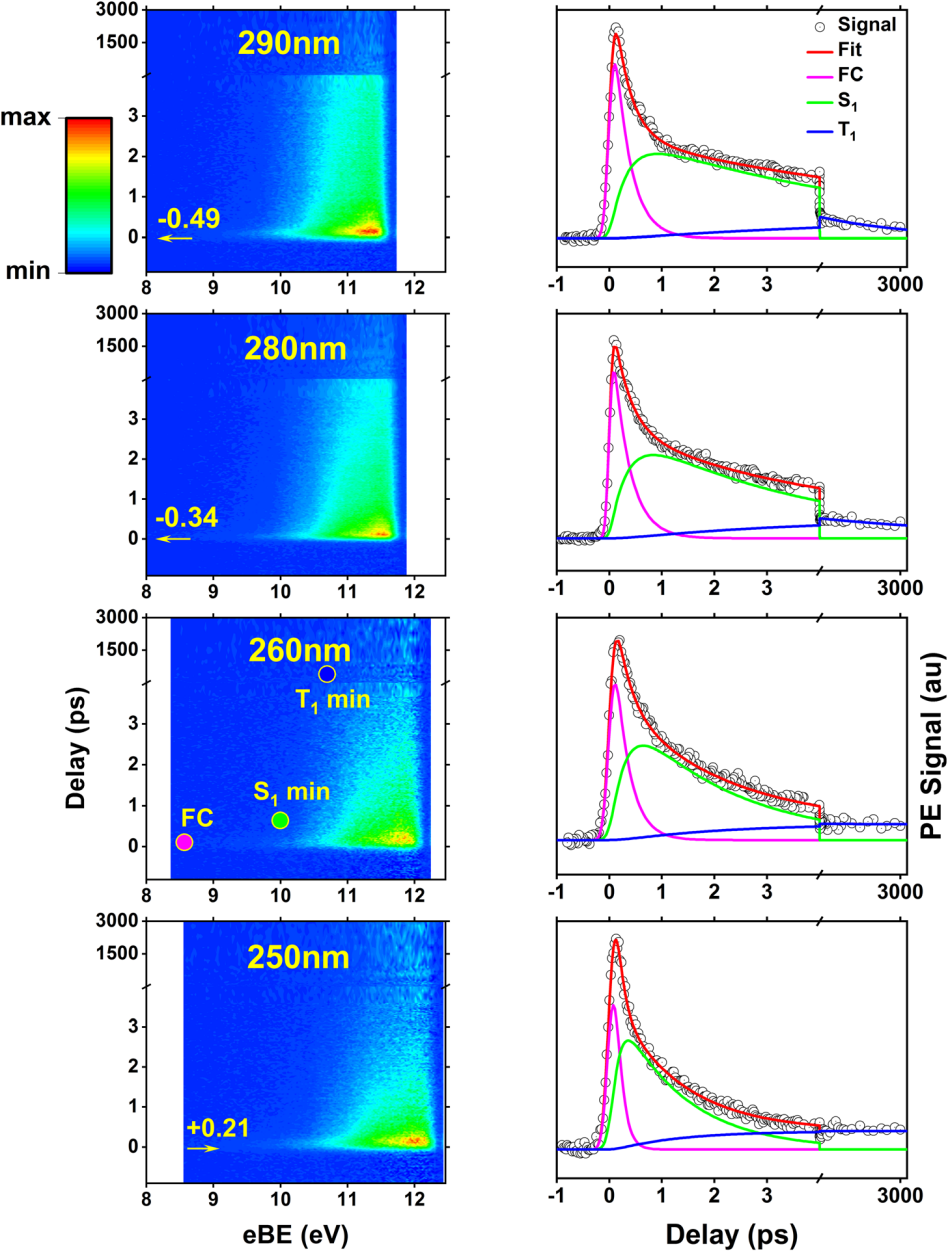
Time-resolved photoelectron spectra of thiol 2TC (first column) plotted as colormaps with pump–probe delays and photoelectron binding energies (eBE) along the vertical and horizontal axis, respectively. The color scheme represents maximum to minimum signal intensity with red, yellow, green, to blue. An axis break is placed at 4 ps pump–probe delay to better visualize the ultrafast region where significant shifts in the photoelectron spectrum occur. The second column shows energy-integrated time traces (photoelectron signal *vs.* delay) with the individual exponential fit components extracted from global analysis. Each row corresponds to a different pump wavelength, 290 nm, 280 nm, 260 nm, and 250 nm, but the same 330 nm two-photon ionization. Additional TRPES at 300 nm, 270 nm, and 243 nm photoexcitation are provided in Fig. S9 of the ESI.† The colored dots superimposed onto the 260 nm TRPES colormap approximate the binding energy range (*i.e.* TBEs) where photoelectron bands from ionization at the FC region and the excited state minima are expected. The amount of vibrational excitation depends on the pump wavelength, leading to a shift of the TRPES signal along eBE axis that corresponds to the difference in photon energy. The arrows and estimated shifts in the TRPES plots are relative to the 260 nm TRPES.

The time-resolved photoelectron spectra of 2TC photoexcited at various wavelengths across the first and second absorption bands (Fig. 2, black arrows) are shown in Fig. 4. For a direct comparison to TBEs from theory, the TRPES spectra are plotted with an electron binding energy (eBE) axis which is calculated as the total photon energy minus the measured electron kinetic energies. While TBE and eBE both refer to the energies of the final vibrationally excited cationic state that the photodynamics are projected onto, TBE refers to a theoretical prediction and eBE is derived from experimental measurements.

For each pump wavelength, significant changes in the photoelectron bands during the first few picoseconds following photoexcitation are clearly visible which are caused by various factors: (1) Koopmann'’s like ionization preferences to different cationic states based on the excited state orbital character, (2) changes in ionization potential along the relaxation path, and (3) vibrational excitation during electronic relaxation that is transferred into the cationic state upon ionization. Taking these factors into account, one can estimate the TBE range where photoelectron bands for ionization from different points along the relaxation path should appear. This is illustrated for the 260 nm TRPES with the superimposed colored dots. At this pump wavelength, 2TC is photoexcited close to its first absorption band maximum, which is directly comparable to the scenario depicted in the theoretical pathways in Fig. 3 and the TBEs in Table S2.† The total photon energy is well above all TBEs and sufficiently captures the excited state photoelectron spectra along the deactivation pathway. The population dynamics of each electronically excited state can be extracted through global lifetime analysis which provides decay time constants (Table 3), and evolution associated spectra (EAS). Applying a sequential decay model with three exponentials, the time traces and decay components in Fig. 4 (right) were obtained; the corresponding EASs are provided in Fig. S9 of the ESI.† For comparison, the fit results based on a parallel decay model with three exponentials are provided in Fig. S10.† The time constants obtained from the fit represent the lifetimes of specific excited states, *i.e.* they measure the population decays of the states which can occur along multiple pathways. A detailed justification for the fitting strategy is also provided in Fig. S11 and S12 of the ESI.† The analysis and interpretation of the photoelectron spectra and time traces of the three sequential decay steps offers insight into the relaxation mechanism.

As the pump wavelength is changed, the same electronic state corresponding to the first absorption band is excited but with different amounts of vibrational excess energy. This excess vibrational energy is transferred to the cation upon ionization and hence shifts the entire TRPES data along the eBE axis. The shift relative to the 260 nm TRPES (see Table S3 in the ESI†) is estimated as the difference in pump photon energy and the amount of this shift is represented by the values and horizontal arrows on the colormap plots.

### Characteristics of photoelectron spectra

During the cross-correlation time (around 0 fs pump–probe delay), vertical photoexcitation into the FC region of the lowest bright ^1^ππ* state launches the excited state dynamics which are projected onto the lowest cationic state through instantaneous vertical ionization with the two-photon probe. As a result, a photoelectron band in the eBE range of the ground state (GS) IE (TBE@S_0 min_ = 8.57 eV, Table S2†) is expected which is indicated by the magenta dot on the 260 nm TRPES colormap and is in line with the observed onset of the EAS around ∼9 eV (Fig. S9†). Photoexcitation is immediately followed by a barrierless motion out of the FC region towards the three S_1_ minima (^1^ππ* and ^1^nπ*, see Fig. 3a, columns 1 and 2), although the minima are indistinguishable with TRPES. Motion out of the FC region is associated with a large (∼1.5 eV) shift in the photoelectron spectrum toward higher eBE within timescales that are too fast to resolve, which produces a broad and rather weak signal at low binding energies around *t* = 0. This shift is due to an increase in ionization energy and vibrational energy gain during relaxation into the S_1_ minima. We note that at all optimized minima, ionization happens through the D_0_ ionization channel, which is of π^−1^ character at the S^π^_1_ (^1^ππ*) minima and of n^−1^ character at the S^n^_1_ (^1^nπ*) minimum due to a switch in ionization correlation. The expected eBE range for ionization from all three S_1_ minima is estimated to be similar, about 10 eV (TBE @ S_1 min_, Table S2 in ESI†). This general eBE range is therefore indicated with a single green dot labeled “S_1_ min” in Fig. 4 and agrees well with the observed shift. From the three S_1_ minima, population can decay back to the ground state through IC (Fig. 3b) or intersystem cross into the triplet manifold and relax into the three T_1_ minima (T^πa,b^_1_ or T^n^_1_, see Fig. 3a). The latter process is associated with another small shift of the photoelectron band toward higher eBE. The computed TBEs for ionization from all T_1_ minima is similar (about 10.7 eV for the T^π^_1_ minima and about 10.4 eV for T^n^_1_ minimum) and is therefore indicated by a single blue dot in Fig. 4, reproducing the experimental observation at long delays. While the broadness of the photoelectron spectral features hampers a clear distinction of the ultrafast, parallel decay processes, further insight into the relaxation paths and mechanism can be gained from dynamical aspects reflected in the excitation energy dependence of the time constants or lifetimes.

### Time trace characteristics

**Fig. 5:**
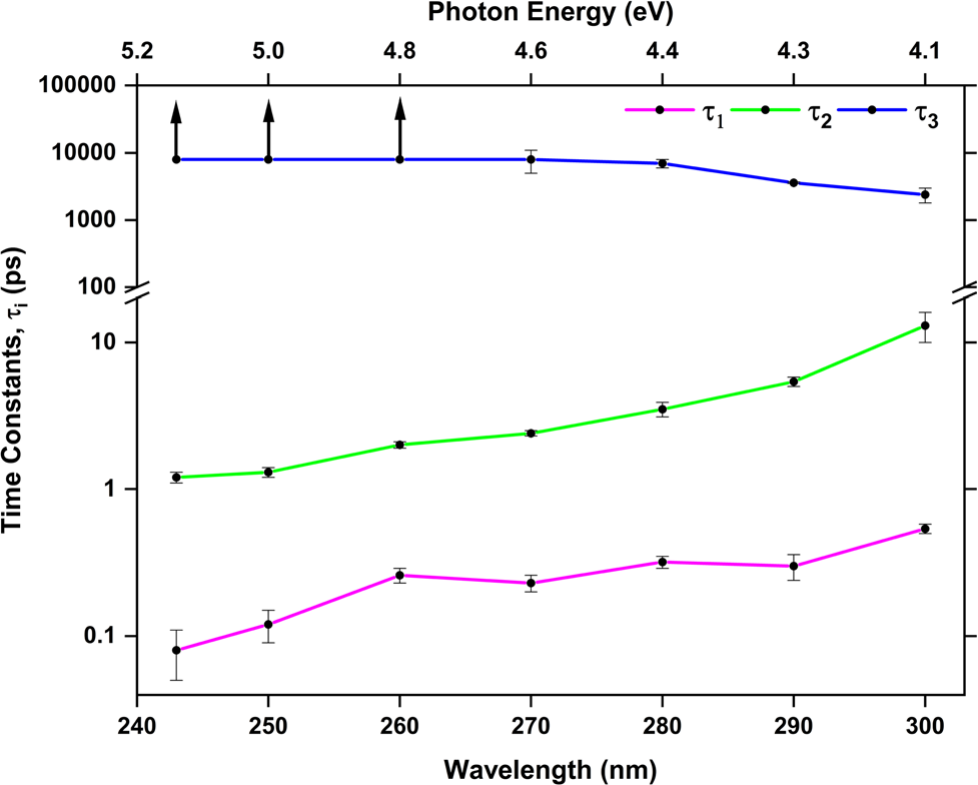
Trend of time constants (*τ*_1_ – magenta, *τ*_2_ – green, and *τ*_3_ – blue) with pump wavelengths. Note the time constant axis is split and both segments are logarithmic. The vertical arrows indicate that the values are greater than 8 ns.

**Fig. 6:**
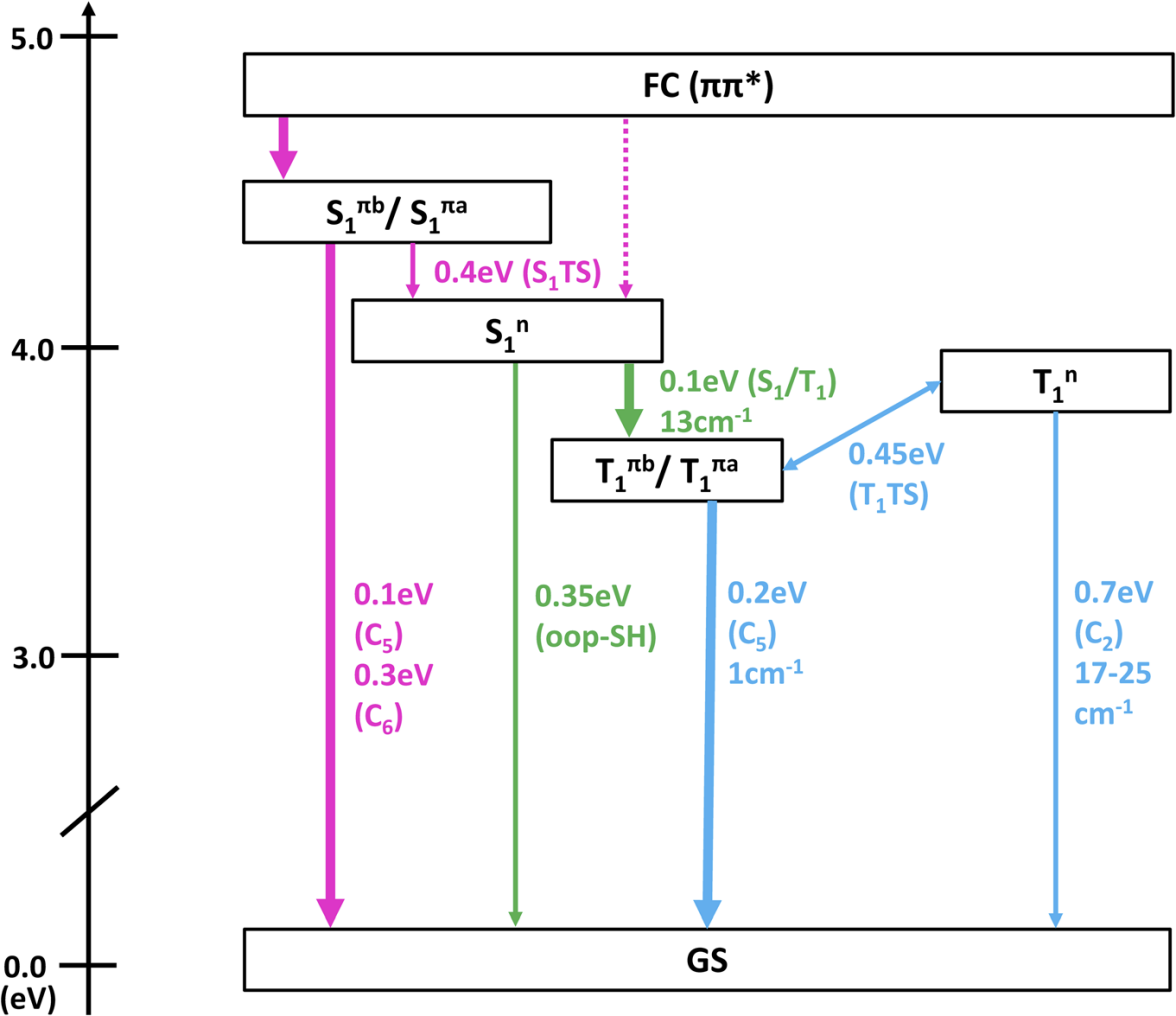
Decay dynamics of gas phase thiol 2TC. The arrows indicate deactivation paths between different states using the same color scheme as the time trace components associated with time constants *τ*_1_ (magenta), *τ*_2_ (green), *τ*_3_ (blue). Barriers (in eV) and SOCs (in cm^−1^) are given as applicable for relevant crossing points (labels in parentheses). The thickness of the arrows indicates the relevance of a process, and the dashed arrow marks a process that is considered negligible. “C_2_”, “C_5_”, and “C_6_” refer to puckering at C_2_, C_5_, and C_6_ atoms. “oop-SH” refers to an out-of-plane distortion of the thiol group. “S_1_TS” and “T_1_TS” refer to transition states on the S_1_ and T_1_, which are characterized by a puckering at the C_6_-atom and an out-of-plane displacement of the thiol group, respectively. The S_1_/T_1_ crossing point involves puckering at the C_2_ atom. These latter displacements are distinct from the relaxation coordinates leading to GS repopulation.

Table 3 collects the recorded excitation energy-dependent time constants or lifetimes, and Fig. 5 visualizes the trend of these lifetimes as a function of the pump wavelengths. The lifetime *τ*_1_, which ranges from about 100 fs to few hundred fs, is related to the fastest IC processes. Although three barrierless relaxation pathways from the FC region to the S_1_ minima are available, intuitively the adiabatic relaxation paths FC → S^π^_1_ (Fig. 3a, column 2) will dominate over IC FC → S^n^_1_ (Fig. 3a, column 1), because the latter requires a switch in orbital character. Continuation from the S_1_ minima toward ground state repopulation is possible for all three pathways. The lifetime *τ*_1_, which captures both the motion out of the FC region to the S^π^_1_ minima and the depopulation of S^π^_1_, shows a decreasing trend with increasing vibrational excitation, *i.e.*, toward shorter pump wavelengths. This behavior is indicative of the presence of a barrier to access the S_1_/S_0_ CIs, with higher vibrational excitation easing barrier crossing. According to the calculations (Fig. 3b), small barriers are expected along each of the IC paths, *i.e.*, 0.1 eV and 0.3 eV along C^b^_5_ and C^b^_6_ (S^πb^_1_ → S_1_/S_0_), respectively, and 0.35 eV along oop-SH (S^n^_1_ → S_1_/S_0_). Relaxation along the adiabatic S^πb^_1_ → S_1_/S_0_ coordinate is driven from the near-planar FC region by a torsion of the C_5_=C_6_ double bond, which leads to ring puckering localized either at the C_5_ or C_6_ atom. At 280 nm and 260 nm excitation, the lifetimes are slightly longer than anticipated (*i.e.*, *τ*_1_ exhibits a stepwise decrease with increasing excitation energy), which may hint at the opening of alternative pathways with higher barriers. For example, at the longest excitation wavelengths IC may be limited to the C^b^_5_ CI but with the additional energy at 280 nm barrier crossing to C^b^_6_ might become feasible as well (0.3 eV barrier). Similarly, S^πb^_1_ → S^n^_1_ IC (Fig. 3a, column 3) which requires surpassing a 0.4 eV barrier may become available, but is less likely, as it involves channeling of vibrational energy from other puckering coordinates into an oop-SH deformation that requires a large amplitude motion of a heavy substituent. Several other pathways with barriers of 0.6 eV and higher might potentially contribute at even shorter excitation wavelengths, which may explain the second discontinuity in *τ*_1_ trend starting at 260 nm. While these interpretations could be taken as speculative, they provide a picture that is consistent with both theory and observations. These processes are visualized as magenta arrows in Fig. 6, although we consider FC (^1^ππ*) → S^π^_1_ → GS as the defining contribution to the lifetime *τ*_1_.

If the system relaxes from the FC region directly into S^n^_1_, the system encounters sizable barriers (0.35 eV or higher) that prevent ultrafast (fs) IC to the ground state and temporarily trap population in S^n^_1_. Furthermore, a low barrier to the S_1_/T_2_ crossing point enables ISC from S^n^_1_ into the triplet manifold, although with low SOC (Fig. 3a, column 4). These factors lead to dynamics on longer, picosecond timescales. The lifetime *τ*_2_ is therefore associated with the population decay of S^n^_1_*via* ISC (green arrow in Fig. 6). The sizable barriers to escape from this minimum are consistent with the significant decrease in *τ*_2_ from 13 ps to 1 ps as vibrational excess energy increases and IC to the ground state become feasible options.

The ns timescale *τ*_3_ is consistent with population trapping in a long-lived excited state. An obvious candidate is the lowest triplet state T_1_ with a high barrier and low SOC for crossing back to the ground state (blue arrows in Fig. 6). We observe a surprising trend to *larger τ*_3_ with decreasing excitation wavelength, suggesting that—unintuitively—an increase in vibrational energy slows down the decay dynamics of the lowest triplet excited state. In principle, higher excitation energies could hypothetically excite higher-lying electronic states that might participate in additional deactivation pathways. However, the dynamics simulations show that the higher excited states S_3_ or S_4_ are short-lived (Fig. S7†). A better explanation for the unexpected trend in *τ*_3_ is given by the presence of a second T^n^_1_ (^3^nπ*) minimum approximately 0.25 eV above the T^πb^_1_ (^3^ππ*) minimum and separated by a 0.45 eV barrier (with respect to the T^πb^_1_ (^3^ππ*) minimum). At shorter excitation wavelengths, *i.e.*, with more vibrational energy, some of T^πb^_1_ (^3^ππ*) population is expected to cross into the T^n^_1_ (^3^nπ*) minimum, from where we expect very slow ISC to the ground state due to the very large barriers needed to reach T_1_/S_0_ crossings from there. Thus, the T^n^_1_ (^3^nπ*) minimum serves to retardate the overall decay of the triplet state to the ground state.

### Effects of tautomerization and thionation in nucleobases

**Fig. 7:**
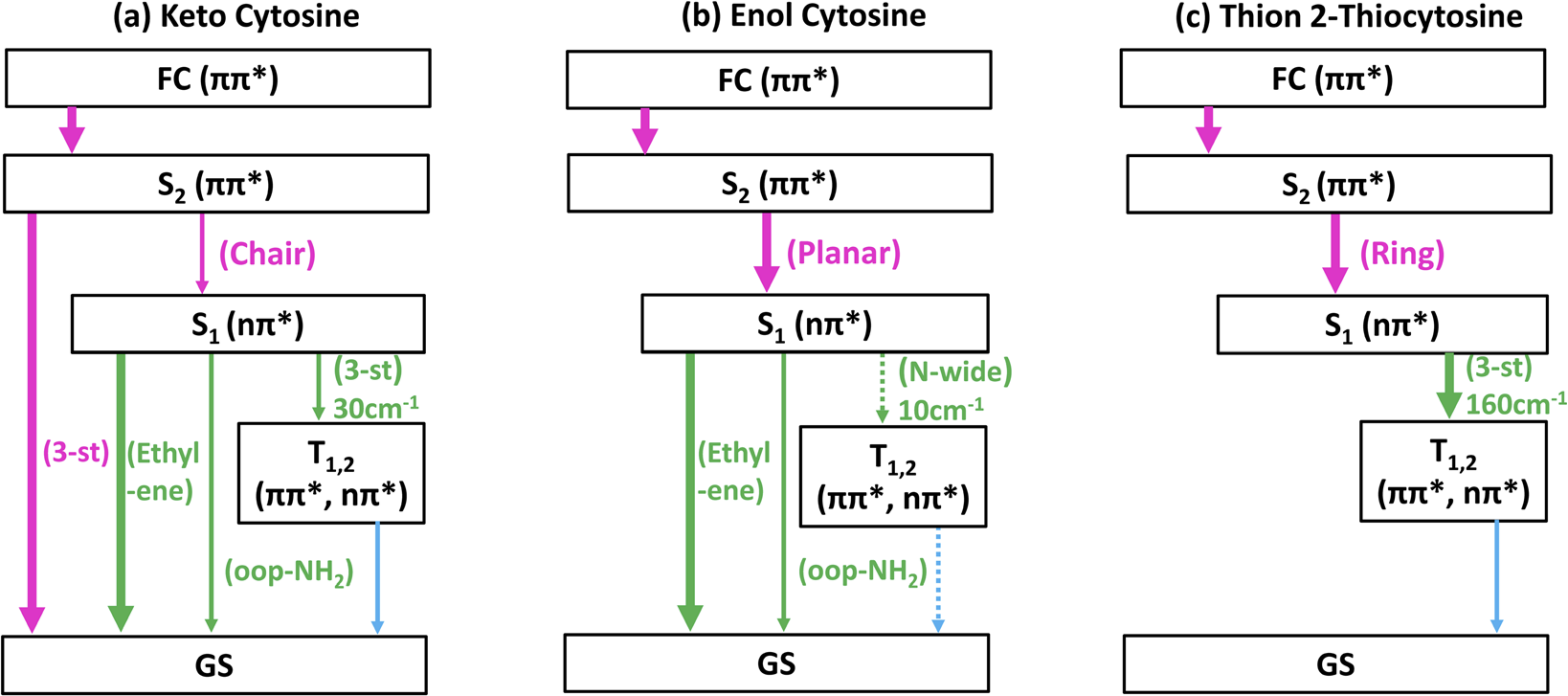
Simplified schemes of the decay dynamics of keto cytosine, enol cytosine, and thion 2TC.^19,25,64^ The population dynamics are color coded as follows: magenta arrows denote decay from the FC region and the S_2_(^1^ππ**) state, green arrows represent decay of the S_1_(^1^nπ**) state, and blue arrows designate decay processes involving triplet states. SOCs (in cm^−1^) are given where applicable. The thickness of arrows indicates relevance of the decay channels with dashed arrows showing processes that are considered negligible. The labels of conical intersections are descriptive of the following coordinates: (a) keto cytosine – chair: chair-like conformational distortion with angling C_4_ and amino group, and C_2_=O stretch, S_2_/S_1_/gs 3st: 3-state near degeneracy characterized by C_5_=C_6_ and C_2_=O stretches, ethylene: “ethylene-like” geometry involving C_5_=C_6_ bond torsion, oop-NH_2_: strong out–out-plane distortion of amino group, and S_1_/T_2_/T_1_ 3-st: 3-state near degeneracy characterized by C_2_=O elongation and C_2_–N_3_ shortening (b) enol cytosine – planar: plane structure resembling ground state minimum, ethylene: “ethylene-like” geometry with C_5_=C_6_ bond torsion, oop-NH_2_: strong out-of-plane deformation of amino group, and N-wide: planar showing large C_6_–N_1_–C_2_ and C_2_–N_3_–C_4_ angles (c) thion 2TC – ring: bond inversion within conjugated double system of pyrimidine ring with increase in ring puckering, and 3st: 3-state near degeneracy characterized by increase in ring puckering.

Further insight into tautomer- and thionation-induced effects on the IC and ISC mechanism in 2TC can be gained through a comparison to keto and enol cytosine as well as thion 2TC, which have been extensively studied in the literature. Schemes of the deactivation process of keto and enol cytosine and thion 2TC based on nonadiabatic dynamics simulation are shown in Fig. 7.^19,25^ In the following paragraphs, we compare the different molecules step by step.

First, we recapitulate keto and enol cytosine. As shown in Fig. 1, keto cytosine possesses a carbonyl group (C_2_=O) and a protonated N atom (N_1_H), whereas enol cytosine exhibits a C_2_–OH motif and unprotonated N atoms. The most likely deactivation pathway from the S_1_ state to the ground state involves the “ethylene-like” CI characterized by twisting of the C_5_=C_6_ bond.^64^ This CI is present in both tautomers, as could be expected from the C_5_=C_6_ bond not being modified by tautomerization. The tautomers differ in their propensity for ISC. In keto cytosine, ISC is an observable side process. It is induced by a S_1_/T_2_/T_1_ three-state crossing that is characterized by an elongated C_2_=O bond, electronic states localized on the carbonyl group (^1^n_O_π*, ^3^n_O_π*, ^3^ππ*), and decent SOCs (30 cm^−1^).^19^ In enol cytosine, ISC is negligible. The blockage of the carbonyl O atom removes the ^1^n_O_π* states and the related three-state crossing. The presence of additional ^1^n_N_π* states does lead to ISC, but only very minor, as the singlet-triplet crossings are less accessible^8^ and the related SOCs are smaller (10 cm^−1^).^19^ Thus, in cytosine, the pyrimidine ring is the source of the main deactivation pathway and the carbonyl group of a side channel. Furthermore, the crossing point between the lowest two triplet states (T_2_ and T_1_) is characterized by pyramidalization of the protonated N_1_ atom in keto cytosine and widening of C_6_–N_1_–C_2_ and C_2_–N_3_–C_4_ angles at unprotonated N_1_ and N_3_ atoms in enol cytosine with the lowest triplet state being expected to intersystem cross to the ground state.

Second, going from keto cytosine to thion 2TC (*i.e.*, replacing the C_2_=O group by C_2_=S) lowers the energy of the thiocarbonyl-centered states (^1^n_S_π*, ^3^n_S_π*, ^3^π_S_π*) significantly. Consequently, these states become the lowest—and dynamics-governing—states. The absorption spectrum associated with singlet ^1^π_s_π* states also red shifts. IC to S_0_ is blocked, as the CIs originating on the pyrimidine ring cannot be reached from the thiocarbonyl-centered low-lying minima. The IC from S_2_ to S_1_ is characterized by bond inversion within the conjugated double bond system of pyrimidine ring. ISC, which is a side channel in keto cytosine, becomes the only relevant channel in thion 2TC and proceeds *via* a three-state near degeneracy of S_1_/T_2_/T_1_.^25^ Due to the energetic proximity of ^1^n_S_π*, ^3^n_S_π*, and ^3^π_S_π*, and the strong SOC (170 cm^−1^) between ^1^n_S_π* and ^3^π_S_π* caused by the localization of the n_S_ → π_S_ transition density on the heavy sulfur atom, ISC is enhanced. As the molecule decays further to S_1_ and triplets (T_2_ and T_1_), ring puckering increases, and the intersystem crossing to ground state is expected. It should be pointed out that these mechanistic details were extracted from *in vacuo* calculations, even though thion 2TC only exists in solution, and should therefore be directly comparable to the present gas-phase study of thiol 2TC.

Third, the present study has shown that enol cytosine and thiol 2TC display strong similarities in their deactivation dynamics. Both undergo efficient decay to the ground state, using the CIs involving the C_5_=C_6_ bond (termed “C_5_” and “C_6_” above and equivalent to the “ethylene-like” CIs in the literature),^8,19^ which is not affected by the thionation. Neither the –OH nor –SH groups are good electron donors, so no electronic states localized on these groups appear, and consequently, no “heavy-atom effect” due to the introduction of the sulfur atom is observed. Interestingly, thiol 2TC still shows some observable ISC, unlike enol cytosine. ISC in thiol 2TC involves the ^1^n_N_π* and ^3^ππ* states, which are also present in enol cytosine. The SOCs between these states are small (10–13 cm^−1^) due to the localization on N atoms rather than the S atom, and barriers limit access to the singlet-triplet crossings with conical intersection S_1_/T_1_ characterized by puckering at C_2_ atom However, the –SH group in thiol 2TC can much more easily rotate in the excited state (see Fig. S3–S6 and S8†) compared to the –OH group in enol cytosine, and the rotation of the –SH group has a subtle effect on the energetics of the ^1^n_N_π*, facilitating easier access to singlet-triplet crossings than in enol cytosine. This is the reason for the increased ISC yield in thiol 2TC compared to enol cytosine. The similarities are more apparent when we compare the time constants of TRPES of 2TC with the previous time-resolved studies at the enol dominant region (∼4.5 eV or ∼260 nm) of cytosine.^18^ The various time-resolved ion yield studies of cytosine at 260 nm pump wavelength give time constants in the range of 120–240 fs and 2.4–3.8 ps.^65–67^ These two time constants are very close to the first (260 fs) and second (2.0 ps) time constants of 2TC TRPES obtained at 260 nm.

From the previous discussion, it is clear that the relaxation and ISC dynamics of cytosine and 2TC are governed by which functional group contributes the lowest-lying electronic states. Here, the pyrimidine ring and the carbonyl group provide states of similar energy, so that in keto cytosine IC and ISC compete to some extent. In enol cytosine and thiol 2TC, the pyrimidine ring alone determines the dynamics, so that IC strongly dominates. The –OH and –SH groups do not significantly contribute to the excited-state electronic characters. In contrast, the thiocarbonyl group introduces very low-lying states, which supersede the dynamics of the pyrimidine ring states. The crossing points that lead to ISC in keto cytosine, enol cytosine, thion 2TC, and thiol 2TC involve C_2_=O bond elongation, angle widening at N_1_ and N_3_, ring puckering, and puckering at C_2_ atom, respectively.

While the present study has focused on an in-depth comparison of the photodynamics of structurally modified cytosines, the conclusions regarding tautomerism and heavy atom effects are conceptually translatable to other nucleobases. Despite different mechanistic details (*i.e.* absence of ethylenic pathways), a similar scenario has been observed for purine thiobases derived from guanine. Gas-phase thiol 6-thioguanosine exhibits an ISC rate (through crossing points that involve distortion of the heterocycle and out-of-plane rotation of the thiol group) that is about 300–1000 times lower than solvated thion 6-thioguanosine, a metabolite of 6-thioguanine, but 80 times higher than enol guanine.^34^ Although the drastic decrease in ISC rate in thiol 6TG is based on a questionable comparison that fails to take excess vibrational excitation into account, the study nevertheless confirms a tautomer dependence of the deactivation dynamics. Similarly, 6-methylthioguanine,^36^ and 6-methylthioinosine and 6-methylguanosine,^35^ where a methyl group models the enol/thiol tautomer, also display significant differences in decay dynamics from their thion forms.

## Conclusion

Time-resolved photoelectron spectroscopy has been employed to investigate the excited-state decay dynamics of photoexcited thiol 2TC in the gas phase. With the help of extensive *ab initio* studies of the deactivation pathways, the following mechanistic details have been extracted. After photoexcitation of thiol 2TC to the FC region, the molecule barrierlessly relaxes toward three S_1_ minima (S^πa^_1_, S^πb^_1_, S^n^_1_). The dominant pathway follows an adiabatic path along the ^1^ππ* state which then internally converts back to the ground state. This motion occurs on timescales of a few hundred fs. Alternatively, if the molecule internally converts to the ^1^nπ* state, it is temporarily trapped in the S^n^_1_ minimum and, with a lifetime of a few ps, can go *via* intersystem crossing into the lowest triplet state, which is long-lived. Although thiol 2TC is structurally similar to enol cytosine, intersystem crossing is slightly enhanced in thiol 2TC, but it is not as significant as in thion 2TC, which has very efficient sub-ps intersystem crossing and a near-unity triplet yield.

From the comparison of keto/enol cytosine and thion/thiol 2TC, we draw the following key conclusion: substituting oxygen with sulfur in 2TC does not inherently alter its photodynamics. The photodynamics changes only when the sulfur is forming a thiocarbonyl group, as only this group introduces low-lying excited states involving excitations from sulfur-centered orbitals. Blocking the thiocarbonyl group by protonation quenches the dominating role of the thiocarbonyl. A thiol group does not provide as easily excitable orbitals as a thiocarbonyl group. Thus, in thiol 2TC, the relaxation dynamics remain predominantly governed by the pyrimidine ring, just as in enol cytosine.

We anticipate that this finding is broadly applicable, extending beyond thio-nucleobases to other thiocarbonyl compounds. The often-cited “heavy-atom effect” actually possesses some interesting subtleties. It is not sufficient to simply introduce a heavy atom anywhere in a molecule to observe the heavy-atom effect on ISC. Rather, the heavy atom must be involved in the orbital transitions that dominate the low-lying electronic excited states.^68^ If the heavy atom is not involved in these transitions, then it effectively remains a spectator that contributes only small inductive effects and might also affect the dynamics through its heavy mass.

These conclusions may have relevant implications for the dynamics of thiobases and other thiocarbonyls in solution and biological scenarios. Since protonation of the thiocarbonyl group effectively suppresses its dominant effect on photophysics, it is reasonable to expect that hydrogen bonding to the thiocarbonyl group has a similar, albeit weaker effect. Hydrogen bonding—which is ubiquitous in nature—to the thiocarbonyl group would affect the energy of the n_S_ and π_S_ orbitals, and thus blueshift the ^1^n_S_π* and ^1^π_S_π* states and the spectrum of thiobases^41^ and other thiocarbonyl compounds^7,69^ (compared to the gas phase or non-protic solvents). If substantial, this blueshift can affect the competition between thiocarbonyl-centered dynamics and backbone-centered dynamics and thus make a thiobase behave more like its parent nucleobase. Control of the ISC time scale in thiocarbonyls through hydrogen bonding has been recently reported in the literature in several studies. In the case of thioformaldehyde (an unstable theoretical model for thiocarbonyls), no ISC is found in gas phase simulations;^69^ however, when solvent is included, ISC to the triplet states is observed within the first 100 fs after excitation, at which point the reorganization of the solvent shell/hydrogen bonds has modified the singlet-triplet gaps so much that ISC stops.^69,70^ In thion 2TC, the lowest-lying triplet state (^3^nπ*) is shown to be stabilized by water, which significantly affects its triplet-to-ground state ISC lifetime.^71^ A similar dependence of the ISC rate on the presence of a solvent has been shown in 2-thiouracil, in which the decay from the triplet to the ground state is on the tens-of-ps time scale in gas phase but on a few-ns time scale in acetonitrile solution with a step-wise dependence on excitation energy.^27^ Finally, the effect of hydrogen bonding has been demonstrated in a thio-coumarin derivative, in which ISC is slowed down due to hydrogen bonding, as the bond blueshifts ^1^n_S_π* state and make it lose its relevance for ISC.^72^

We note that, overall, the interaction of hydrogen bonds and a thiocarbonyl group is inherently complex, as the hydrogen bond network will react to the excitation of the molecule, and thus the solvent-induced shifts of the singlet and triplet states will evolve over time. A comprehensive understanding of this dynamic interplay of thiocarbonyl molecules and its environment requires further studies—both in solution and in the gas phase—across different tautomers to discriminate intrinsic molecular dynamics from environment-induced effects. At the same time, it is of critical importance to understand which thiocarbonyl molecules have ISC mechanisms that are robust against environmental influences (like some thiobases^5,6^) to design applications that rely on a high ISC yield. One such example is the use of thiobases as photosensitizers in photodynamic drug therapies.^5^ To achieve high selectivity and photodynamic efficacy, thiobases can be metabolized into DNA of highly proliferating tumor cells where they are photoactivated to produce highly reactive T_1_ states that directly or indirectly kill the cancer cells.

## Author contributions

SM: conceptualization, formal analysis, investigation, supervision, validation, visualization, writing – original draft, writing – review & editing; IE: investigation; LG: conceptualization, resources, writing – review & editing, funding acquisition; BD: investigation, formal analysis, visualization, validation, writing – original draft, writing – review & editing; SU: conceptualization, investigation, formal analysis, visualization, validation, writing – original draft, writing – review & editing, resources, funding acquisition, supervision.

## Conflicts of interest

There are no conflicts to declare.

## Supplementary Material

SC-OLF-D5SC01442E-s001

## Data Availability

The ESI† contains substantial details on the quantum chemical calculations and fitting of experimental data. The raw experimental data is saved in a custom format and will be shared upon request.
