# Supplementary material for: Tautomer aspects in the excited-state dynamics in 2-thiocytosine: intersystem crossing in the absence of the thiocarbonyl group
Source: Chem Sci. 2025 Jul 23;16(33):15015–28. doi: 10.1039/d5sc01442e (PMC12284813; doi:10.1039/d5sc01442e)
Supplement: SC-016-D5SC01442E-s001 [file SC-016-D5SC01442E-s001.pdf]

Supplementary Information (SI) for Chemical Science.
This journal is © The Royal Society of Chemistry 2025

Tautomer aspects of excited-state dynamics in 2-thiocytosine: Intersystem crossing in the absence of the thiocarbonyl group

Bijay Duwal,[1] Isabel Eder,[2] Leticia González,[2,3] Sebastian Mai,[2,*] and Susanne Ullrich[1,*]

[1] Department of Physics and Astronomy, University of Georgia, Athens, Georgia 30602, USA.
[2] Institute of Theoretical Chemistry, Faculty of Chemistry, University of Vienna, Währinger Straße 17, 1090, Vienna, Austria.
[3] Vienna Research Platform on Accelerating Photoreaction Discovery, University of Vienna, Währinger Straße 17, 1090, Vienna, Austria.

ORCIDs: BD (0009-0003-6475-9948), IE (0009-0009-8030-5974), LG (0000-0001-5112-794X), SM (0000-0001-5327-8880), SU (0000-0002-1828-2777)

Email: sebastian.mai@univie.ac.at, ullrich@uga.edu

SUPPORTING INFORMATION

Contents

S1. Electronic transitions, excitation energies, energy scheme, and ionization energies 2
S2: Complete path segments and structure of critical points 5
S3: TRPES analysis 12
S4: Fit equation 14
S5: Justification of fits 15
S6: Cartesian co-ordinates of the critical points 16

S1. Electronic transitions, excitation energies, energy scheme, and ionization energies

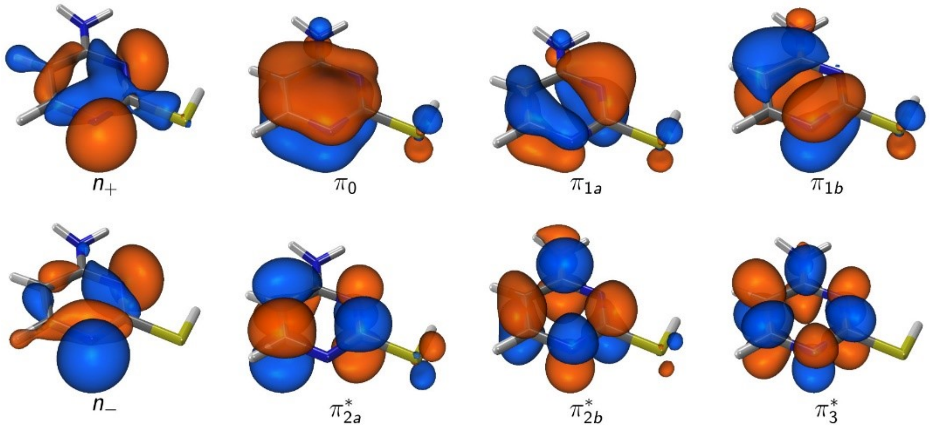

Figure S1 CAS (10,8) orbitals for singlets at the S_0 ^{b} minimum geometry.

Table S1 Vertical excitation energies, oscillator strengths, and orbital characters of singlet states and selected doublet and triplet states computed at the XMS-CASPT2(10,8)/ANO-R1 levels of theory. See Figure S1 for the referenced orbitals.

	**XMS-CASPT2(10,8)/ANO-R1**		
**States**	Vertical Excitation Energy (eV)	Oscillator Strength	Orbital characters
**S_0**	0.00	—	90% closed shell
**S_1**	4.78	0.083	41% $\pi_{1b}\pi^*_{2a}$ minus 29% $n_-\pi^*_{2a}$
**S_2**	4.80	0.049	24% $\pi_{1b}\pi^*_{2a}$ plus 50% $n_-\pi^*_{2a}$
**S_3**	5.20	0.006	78% $n_-\pi^*_{2b}$
**S_4**	5.83	0.076	20% $\pi_{1a}\pi^*_{2a}$ plus 49% $\pi_{1b}\pi^*_{2b}$
**S_5**	6.40	0.004	69% $n_+\pi^*_{2a}$
**S_6**	6.56	0.628	68% $\pi_{1a}\pi^*_{2b}$
**S_7**	6.75	0.005	69% $n_+\pi^*_{2b}$
**S_8**	7.55	0.108	16% $\pi_0\pi^*_{2a}$ plus 28% $\pi_{1a}\pi^*_{2a}$
**S_9**	8.24	0.040	28% $\pi_0\pi^*_{2b}$ plus 14% $\pi_{1b}\pi^*_{2b}$
**D_0**	8.56	—	84% π^{-1}_{1b}
**D_1**	8.98	—	78% n^{-1}_-
**D_2**	9.86	—	83% π^{-1}_{1a}
**D_3**	10.52	—	72% n^{-1}_+
**D_4**	12.00	—	60% π^{-1}_0
**T_1**	4.25	—	25% $\pi_{1b}\pi^*_{2b}$ plus 47% $\pi_{1b}\pi^*_{2a}$
**T_2**	4.55	—	44% $\pi_{1b}\pi^*_{2b}$ minus 37% $\pi_{1b}\pi^*_{2a}$

**T_3**	4.63	—	81% $n\_\pi^*_{2a}$
**T_4**	4.98	—	77% $n\_\pi^*_{2b}$

The energy scheme for photoexcitation, decay dynamics, and photoionization from relevant states, which is used in the interpretation of experimental TRPES data, is shown in Figure S2.

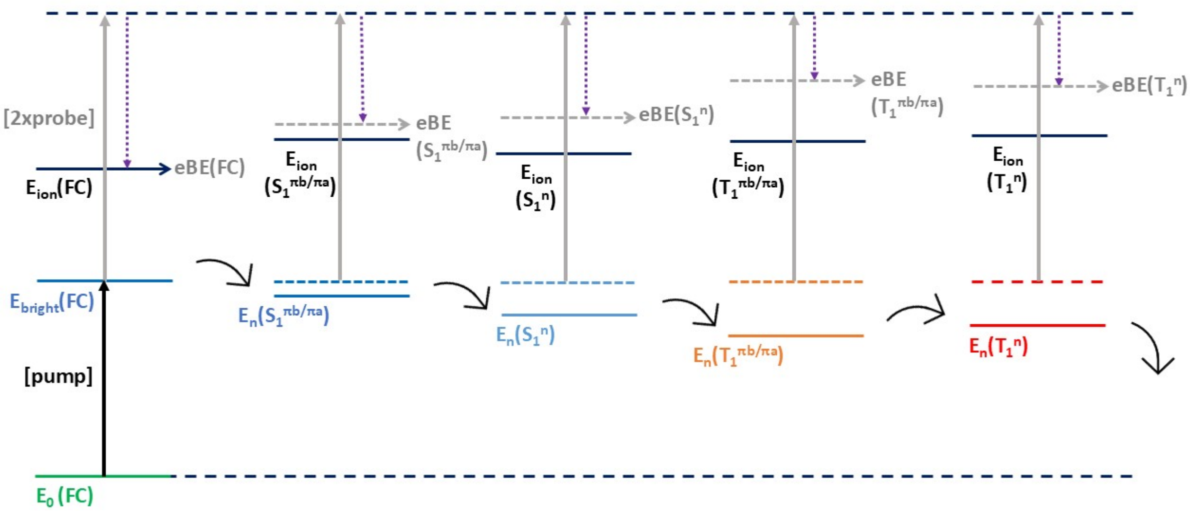

Figure S2 Energy diagram of thiol 2-thiocytosine and schematic of the TRPES technique. The solid horizontal lines visualize the energies of the neutral ground (E_0) and excited states (E_n) and cationic ground state (E_{ion}) in the FC region and at the geometries of relevant minima. The black and grey vertical arrows represent the photon energy of the pump and two-photon probe respectively. The dashed horizontal lines track the vibrational energy gain during electronic relaxation and its transfer to the cation during ionization. The photoelectron kinetic energy, which corresponds to the difference in total photon energy and the energy of the vibrationally excited cation, is shown as purple dotted downward directed arrows. eBEs are the electron binding energies.

$$eBE = \underbrace{E_{pump} + E_{probe}}_{total\ photon\ energy} - \underbrace{eKE}_{photoelectron\ kinetic\ energy} \qquad (1)$$

Equation 1 is used to convert the energy axis of the TRPES data from measured photoelectron kinetic energy (eKE) to electron binding energy (eBE). eBE is the total photon energy of the pump (E_{pump}) and probe (E_{probe}) minus the eKE. This is visualized in an energy scheme in Figure S2. eBE is used throughout the manuscript when referring to this experimental parameter but is comparable to the theoretical total binding energy (TBE) which is defined as below.

$$TBE(R_n) = \underbrace{E_{bright}(FC) - E_0(FC)}_{E_{exc}(FC)} + \underbrace{E_{ion}(R_n) - E_n(R_n)}_{vertical\ IE(R_n)} = \underbrace{E_{ion}(R_n) - E_0(FC)}_{adiabatic\ IE(R_n)} + \underbrace{E_{bright}(FC) - E_n(R_n)}_{E_{vib}\ gain\ (R_n)} \qquad (2)$$

Equation 2 is used to estimate the TBE for ionization from critical points along the theoretical relaxation pathways. This can also be visualized in the energy scheme in Figure S2 where TBE corresponds to the energy difference between E_0(FC) and the energy of the vibrationally excited cationic state at a specific geometry. Critical points that are considered in the analysis are the Franck-Condon region (FC), which corresponds to the geometry of the neutral ground state minimum, and the geometries of the neutral excited state minima. R_n refers to the nuclear geometry of a specific critical point; energies correspond to the initially excited bright state E_{bright}(FC) at the FC geometry, the minimum energy of the neutral ground

state E_0(FC)), and energies $E_n(R_n)$ or $E_{ion}(R_n)$ at critical points of geometry R_n for the neutral excited or cationic state, respectively. All energies were calculated with XMS-CASPT2(10,8)/ANO-R1. Excitation energies, vibrational energy gain during electronic relaxation, adiabatic ionization energies, and the estimated TBEs are tabulated in Table S2. These TBE estimates are based on a simplistic picture that assumes Koopmans like ionization correlations and a propensity for transfer of vibrational excitation to the cation upon photoionization. They provide a rough approximation of the eBE range in the experimental TRPES where photoelectron signals for ionization from specific critical points are expected to occur. This allows us to correlate shifts in the experimental TRPES with points on the excited state potential energy surfaces that are accessed during the relaxation process.

Table S2 Estimation of total binding energies (TBE) where photoelectron signals from different geometries are expected. Excited state energies, vibrational energy gain during electronic relaxation, and adiabatic ionization energies are calculated at different geometries based on XMS-CASPT2(10,8)/ANO-R1.

Geometry	Excited State Energy (eV)	Vibrational Energy (eV)	Adiabatic Ionization Energy (eV)	TBE (eV) = Ionization Energy + Vibrational Energy
**S_0 min (a)**	0.00	—	8.57	8.57
**S_0 min (b)**	0.01	—	8.56	8.56
**S_1 FC (ππ*)**	4.78	—	8.57	8.57
**S_1 min (ππ* b)**	4.41	0.37	9.51	9.88
**S_1 min (ππ* a)**	4.39	0.39	9.80	10.19
**S_1 min (nπ*)**	4.13	0.65	9.42	10.07
**T_1 min (ππ* b)**	3.58	1.20	9.50	10.70
**T_1 min (ππ* a)**	3.57	1.21	9.47	10.68
**T_1 min (nπ*)**	3.85	0.93	9.49	10.42

Since excitation at 260nm (4.77eV) is closest to the maximum of the UV-vis spectrum (4.78eV), we assume, for the purpose of TRPES interpretation, that this corresponds to the FC region. The 260nm TRPES is interpreted based on the estimated TBEs in Table S2 which allows us to confirm the excited states participating in the relaxation process. Assuming that the mechanism remains the same, for other excitation wavelengths, we simply expect a shift of the entire TRPES that corresponds to the difference in pump photon energy (i.e. a difference in vibrational excitation). These expected shifts of the entire TRPES at other pump wavelengths along the eBE axis are estimated with respect to the 260nm TRPES and collated in Table S3.

Table S3 Expected shift of entire TRPES along the eBE axis at different pump wavelengths.

Pump wavelength (nm)	Photon energy (eV)	Expected shift in eBE (eV)
**300nm**	4.14	-0.63
**290nm**	4.28	-0.49
**280nm**	4.43	-0.34
**270nm**	4.60	-0.17
**260nm**	4.77	~0
**250nm**	4.98	+0.21

**243nm**	5.10	+0.33

S2. Complete path segments and structure of critical points

The following figures show *all* the path segments investigated in this work. In comparison to Figure 3 in the main text, they also present all path segments related to the “a” rotamer structures and all path segments from S_1/S_0 and T_1/S_0 crossing points to the S_0 minima.

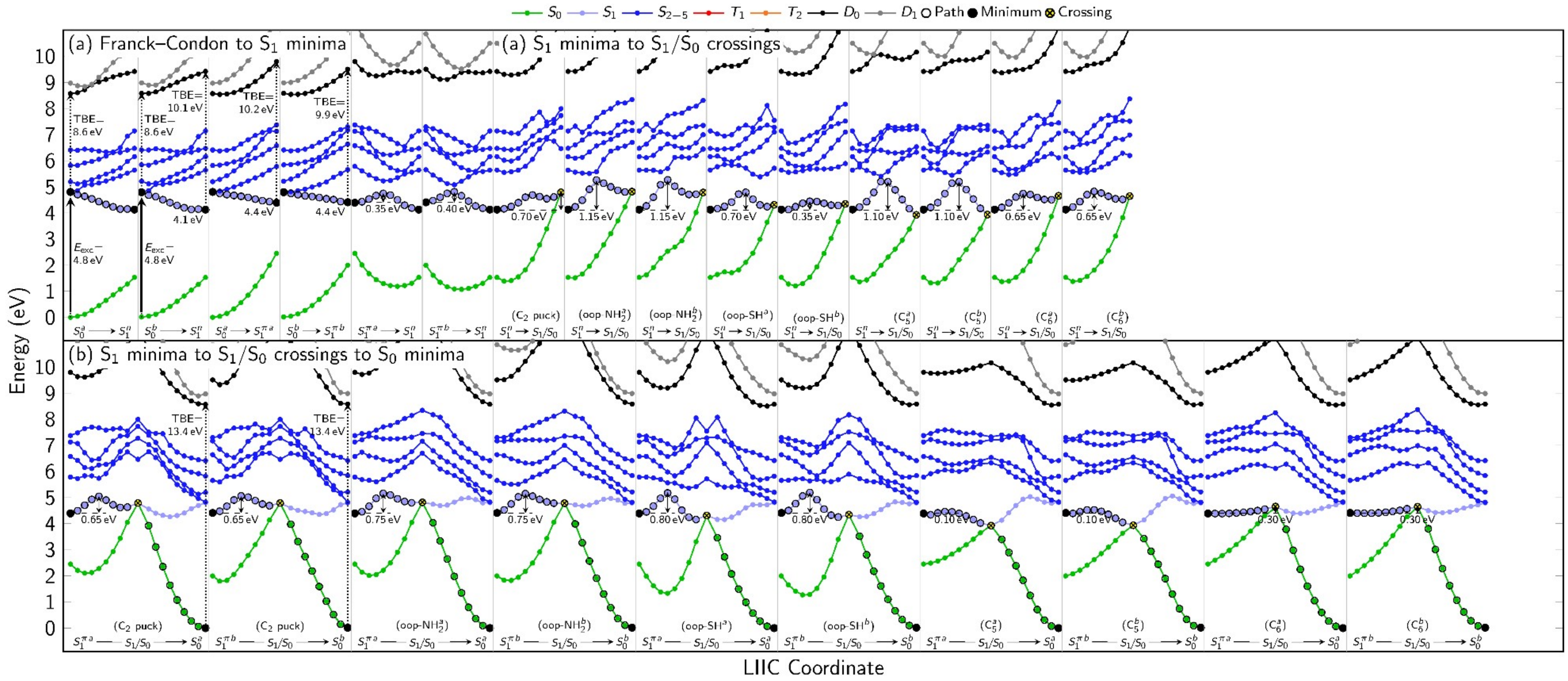

Figure S3 Linear interpolation (in internal coordinates) of singlet-only potential energy surfaces connecting all critical points of the neutral states at the XMS-CASPT2(10,8)/ANO-R1 level of theory studied in this work.

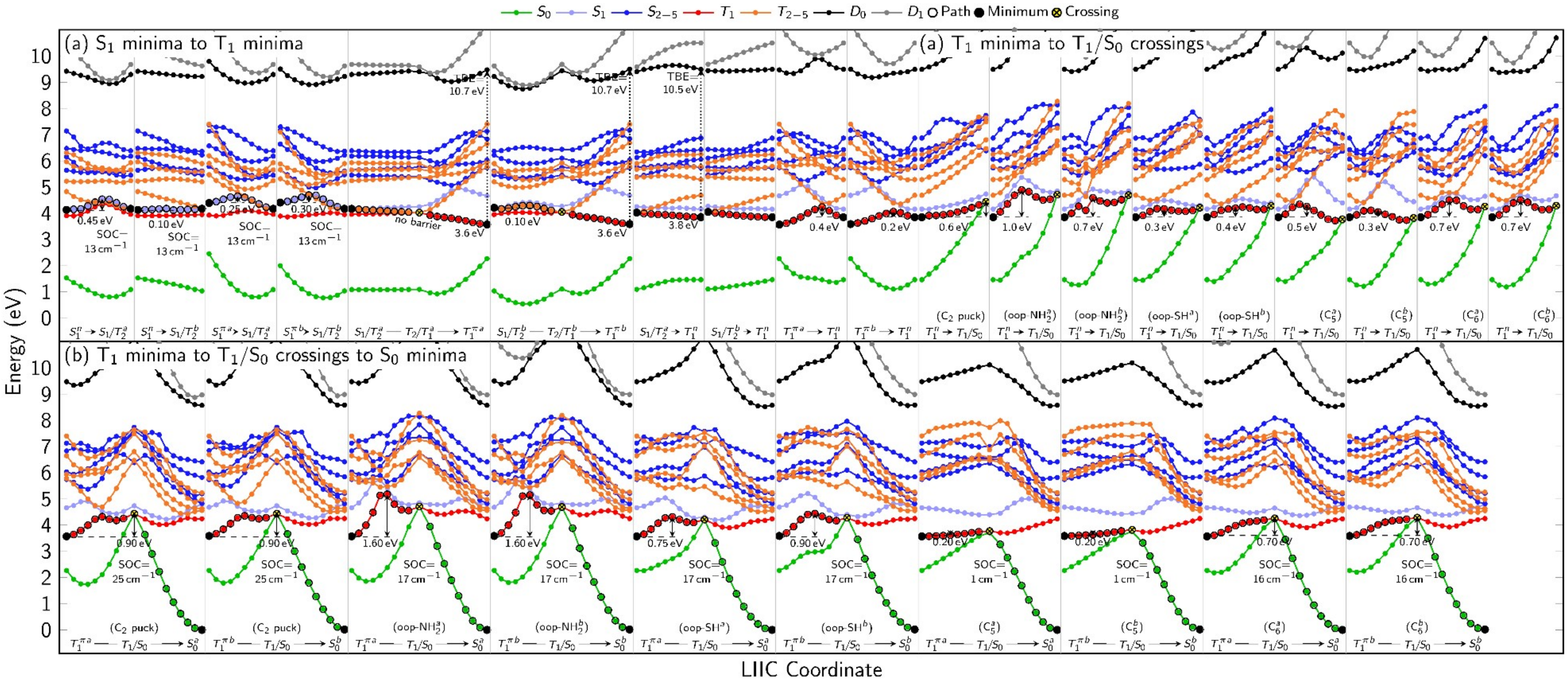

Figure S4 Linear interpolation (in internal coordinates) of singlet and triplet potential energy surfaces connecting all critical points of the neutral states at the XMS-CASPT2(10,8)/ANO-R1 level of theory studied in this work.

The following two figures show all the path segments investigated and the structures of all 29 presented critical points.

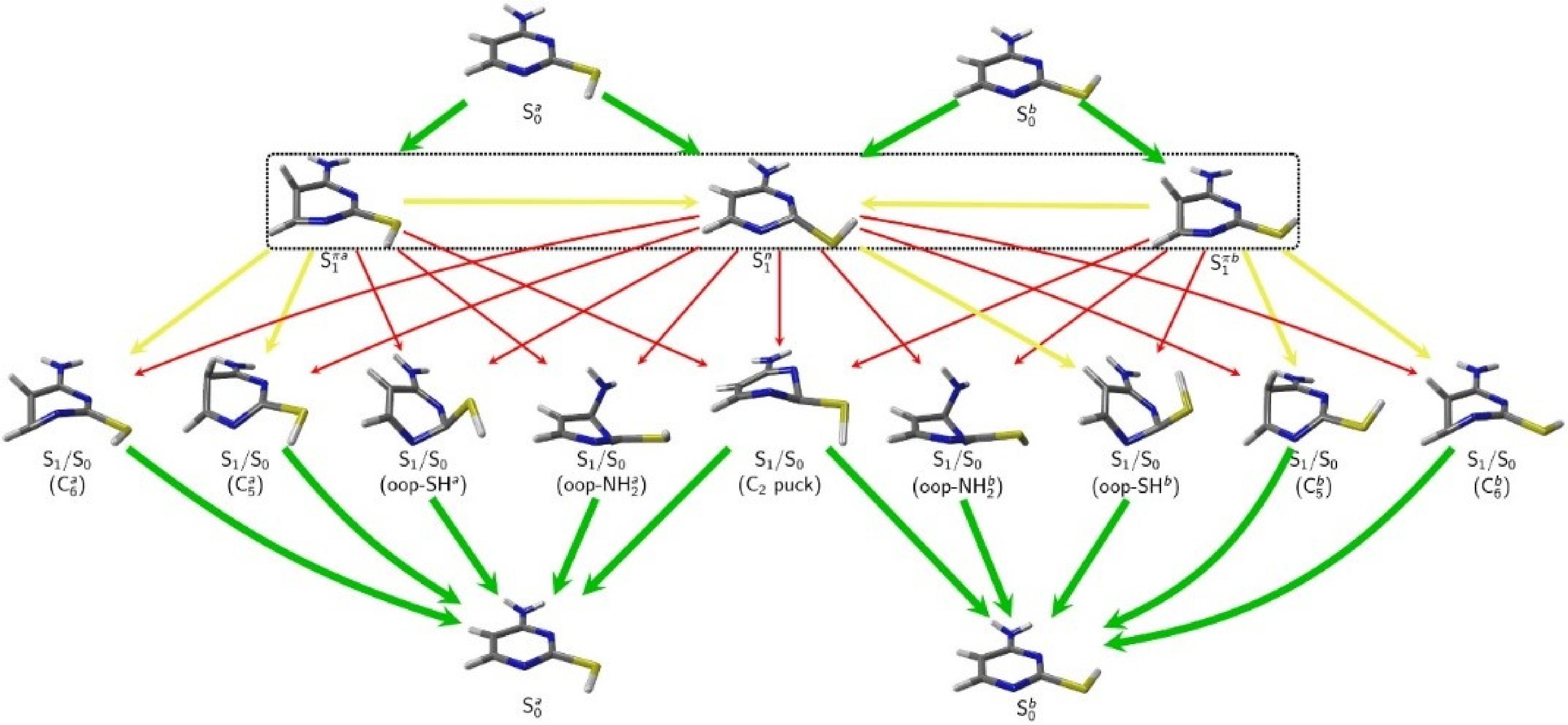

Figure S5 All investigated path segments and structures of all singlet-only critical points

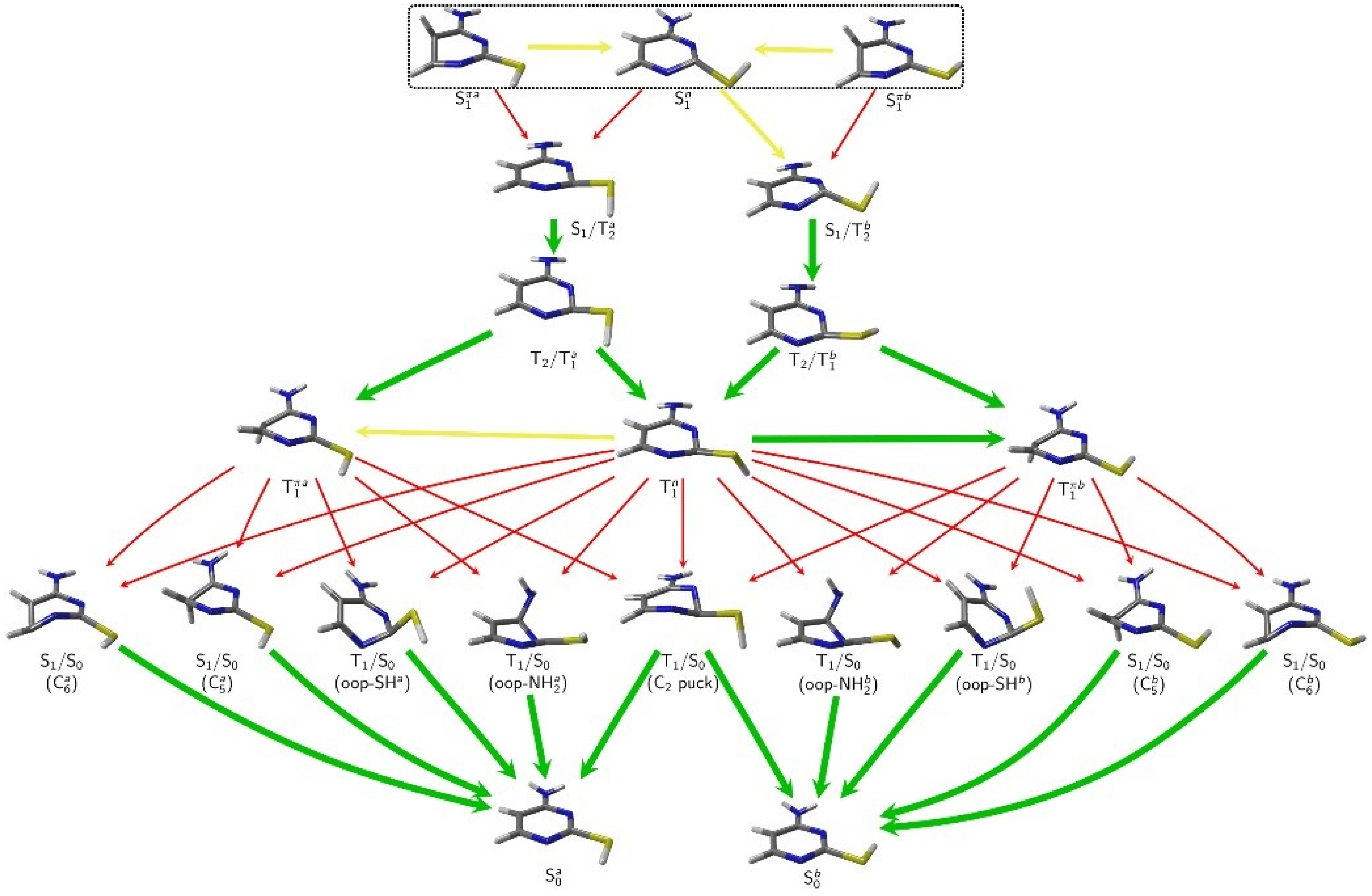

Figure S6 All investigated path segments and structures of all singlet-triplet and triplet critical points

Table S4 Important geometrical parameters of 2TC at the optimized critical points.

Geometry	$R(C_5{=}C_6)$ (Å)	$R(N_3{=}C_4)$ (Å)	$R(N_1{=}C_2)$ (Å)	$R(C_2\text{-}S_8)$ (Å)	Puckering amplitude (Å)	Boeyens classification	Dihedral (N_1-C_2-S_8-H_{13}) (degree)
S_0 min a	1.34	1.35	1.39	1.76	0.01	$^4T_2$	0.2
S_0 min b	1.34	1.35	1.39	1.76	0.01	$^4T_2$	179.9
S_1 min nπ*	1.38	1.32	1.41	1.77	0.17	^2E	-116.3
S_1 min ππ* a	1.36	1.38	1.50	1.75	0.43	E_6	3.0
S_1 min ππ* b	1.36	1.38	1.49	1.75	0.42	E_6	175.8
T_1 min ππ* a	1.36	1.32	1.49	1.75	0.41	$^6S_5$	2.7
T_1 min ππ* b	1.36	1.32	1.49	1.75	0.41	E_5	-175.5
T_1 min nπ*	1.39	1.31	1.40	1.77	0.18	$^1S_2$	134.5
S_1T_2 MECP a	1.40	1.32	1.42	1.76	0.19	E_2	55.3
S_1T_2 MECP b	1.39	1.32	1.42	1.77	0.17	^2E	-167.8
T_2T_1 MECI a	1.41	1.31	1.42	1.77	0.10	E_2	52.8
T_2T_1 MECI b	1.39	1.31	1.43	1.77	0.04	$^3S_2$	173.6
S_1S_0 MECI C_2 puck	1.45	1.36	1.38	1.77	0.68	E_2	87.6
S_1S_0 MECI oop-NH_2 a	1.29	1.50	1.35	1.74	0.54	E_3	-11.3
S_1S_0 MECI oop-NH_2 b	1.29	1.50	1.36	1.74	0.56	E_3	165.8
S_1S_0 MECI oop-SH a	1.47	1.30	1.37	1.80	0.54	$^2S_1$	69.5
S_1S_0 MECI oop-SH b	1.46	1.30	1.37	1.79	0.56	$^2S_1$	-96.2
S_0S_1 MECI C_5 a	1.38	1.35	1.49	1.74	0.57	^5E	-2.9
S_0S_1 MECI C_5 b	1.38	1.35	1.49	1.74	0.57	^5E	175.5
S_0S_1 MECI C_6 a	1.39	1.39	1.50	1.75	0.69	$^1S_6$	-1.2
S_0S_1 MECI C_6 b	1.39	1.38	1.50	1.74	0.68	$^1S_6$	-179.7
S_0T_1 MECP C_2 puck	1.46	1.34	1.38	1.79	0.55	E_2	107.7
S_0T_1 MECP oop-NH_2 a	1.29	1.46	1.37	1.74	0.53	E_3	-13.0
S_0T_1 MECP oop-NH_2 b	1.29	1.46	1.37	1.74	0.54	E_3	160.7
S_0T_1 MECP oop-SH a	1.42	1.30	1.37	1.81	0.51	$^2S_1$	73.0
S_0T_1 MECP oop-SH b	1.41	1.31	1.36	1.79	0.53	$^2S_1$	-84.1
S_0T_1 MECP C_5 a	1.36	1.32	1.48	1.75	0.57	E_5	4.2
S_0T_1 MECP C_5 b	1.35	1.33	1.47	1.75	0.55	E_5	-176.7
S_0T_1 MECP C_6 a	1.39	1.39	1.47	1.75	0.67	$^1S_6$	-4.9
S_0T_1 MECP C_6 b	1.39	1.40	1.47	1.74	0.67	$^1S_6$	175.9

Boeyens classifications: C: chair, H: half-chair, E: envelope, T: twist, S: screw-twist, B: boat. Subscripts (superscripts) indicate indices of atoms below (above) the molecular plane. For example, in $^4T_2$, atoms 1, 3, 5, and 6 form a plane, atom 2 is below this plane and atom 4 is above this plane. Note that the puckering amplitude indicates how strong the ring deformation is. The two S_0 minima are effectively planar, although they are classified as $^4T_2$ conformers.

To investigate the early deactivation dynamics from the bright initial singlet states to the S_1 state and to obtain guess geometries to optimize the various critical geometries, we performed surface hopping simulations. The trajectories were simulated using the SHARC package with ORCA 5.0.4, employing the BP86-D3BJ functional, the def2-SVP basis set, and the def2/J auxiliary basis. The initial conditions were sampled from the ground state harmonic oscillator Wigner distribution: 250 initial conditions at 0K and 350 initial conditions at 500K. All initial conditions were excited in two energy windows: 4.0-4.4eV and 4.4-4.8eV. This provided four sets of trajectories. A total of 216 trajectories were launched and 181 were included in the final analysis (59 at 0K and 4.0-4.4eV; 34 at 0K and 4.4-4.8eV; 33 at 500K and 4.0-4.4eV; 55 at 500K and 4.4-4.8eV). The remaining trajectories were excluded due to total energy conservation issues, but these generally occurred after the initial decay dynamics and thus do not affect the investigation of the decay from the higher singlet states. Trajectories were run for 1000 fs using steps of 0.5 fs. The electronic wave function was propagated using wave function overlaps using the local diabatization technique. An energy-based decoherence correction and rescaling of the full velocity vector after a hop was employed.

Figure S7 shows the electronic populations obtained from the simulations. It can clearly be seen that the initial population is distributed over the different singlet states. In the lower excitation window (a-b), 15-30% of the population starts in the S_1, but in the higher excitation window only few % do so. The lifetimes of the higher singlet states are generally very short, such that after 5-30fs the S_1 is the most populated singlet state, independent of the initial temperature or excitation energy. Within this short time, no appreciable triplet population builds up.

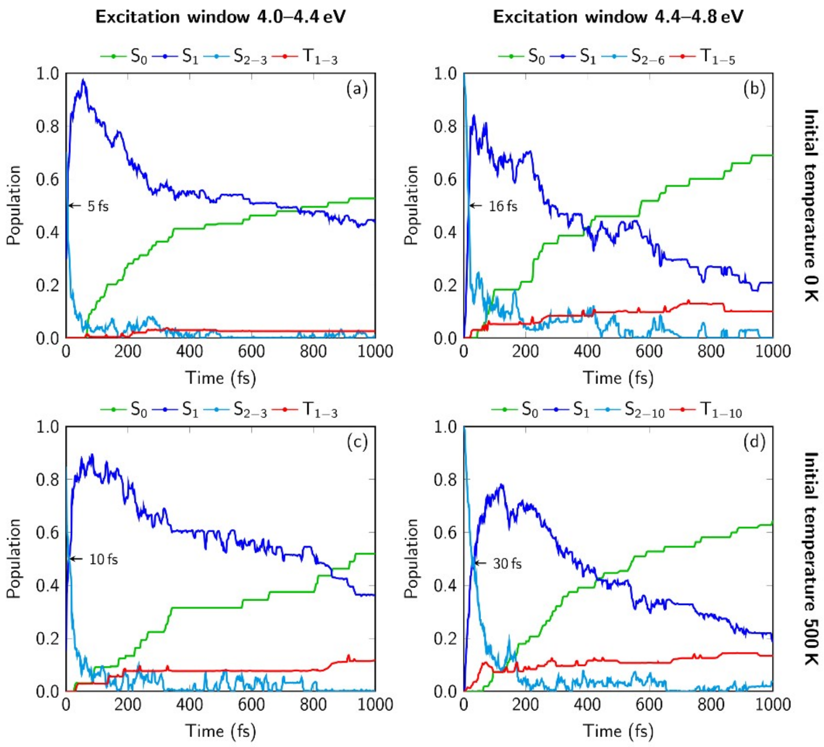

Figure S7 Electronic populations of 2TC from SHARC simulations using different initial temperatures (a-b: 0K, c-d: 500K) and excitation energies (a,c: 4.0-4.4eV, b,d: 4.4-4.8eV). Note the different number of states included in the simulations depending on the initial conditions. The labeled arrows indicate the time it takes for the S_1 population to equal the population of all other singlet states.

Figure S8 shows overlays of all nuclear coordinates of all time steps of all trajectories of the four simulation sets. It can be nicely seen that the pyrimidine ring is very rigid. The most mobile parts of the molecule are the amino group hydrogens and particularly the thiol hydrogen atom. It can clearly be seen that the thiol group can freely rotate in the excited state. However, as seen from the PES scans in Figures S3 and S4, the thiol rotation angle hardly affects the dynamics. Figure S8 shows also that the thiol hydrogen atom is not prone to dissociation. Among all 181 trajectories, only one exhibited a S-H distance of more than 3 Angstrom, but the hydrogen atom returned after about 80fs, indicating that the bond length increase was not driven by a repulsive potential.

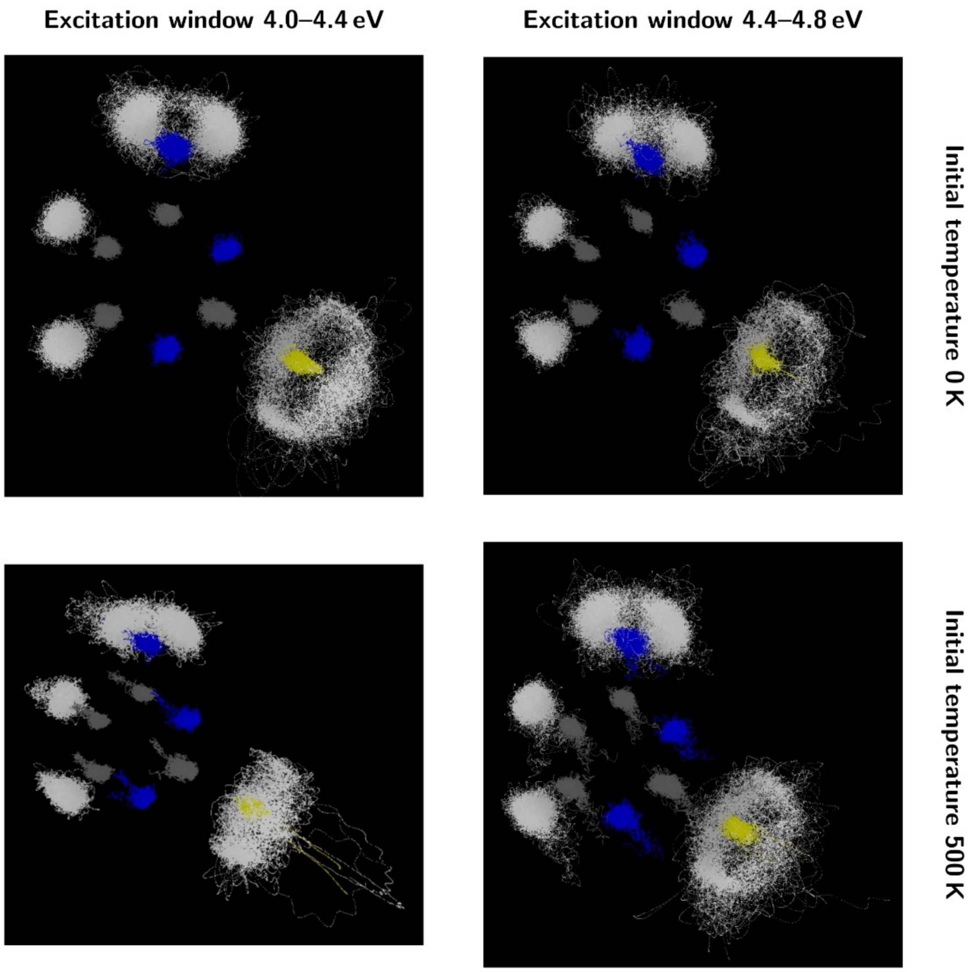

Figure S8 Overlay of all time steps of all trajectories from the four SHARC simulation sets.

S3. TRPES analysis

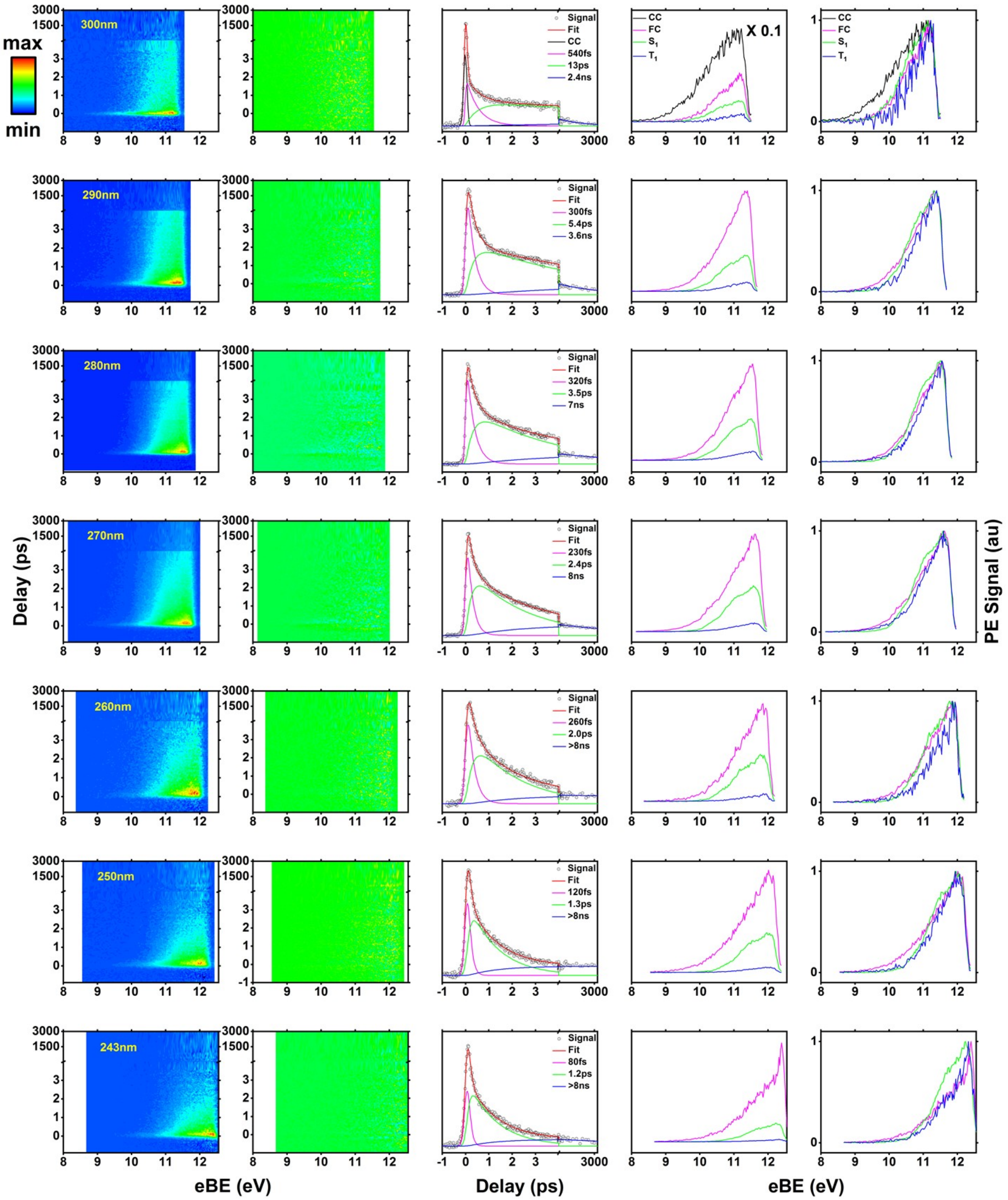

Figure S9 TRPES analysis of 2TC using a three-exponential sequential decay model. Each row corresponds to the TRPES data at a specific pump wavelength. From left to right, the columns show: the TRPES signal, the residuals of the global fit, the energy-integrated time traces, the evolution associated spectra (EAS), and the normalized EAS. The EAS of the ultrafast time constant at 300nm is reduced by a factor of 0.1.

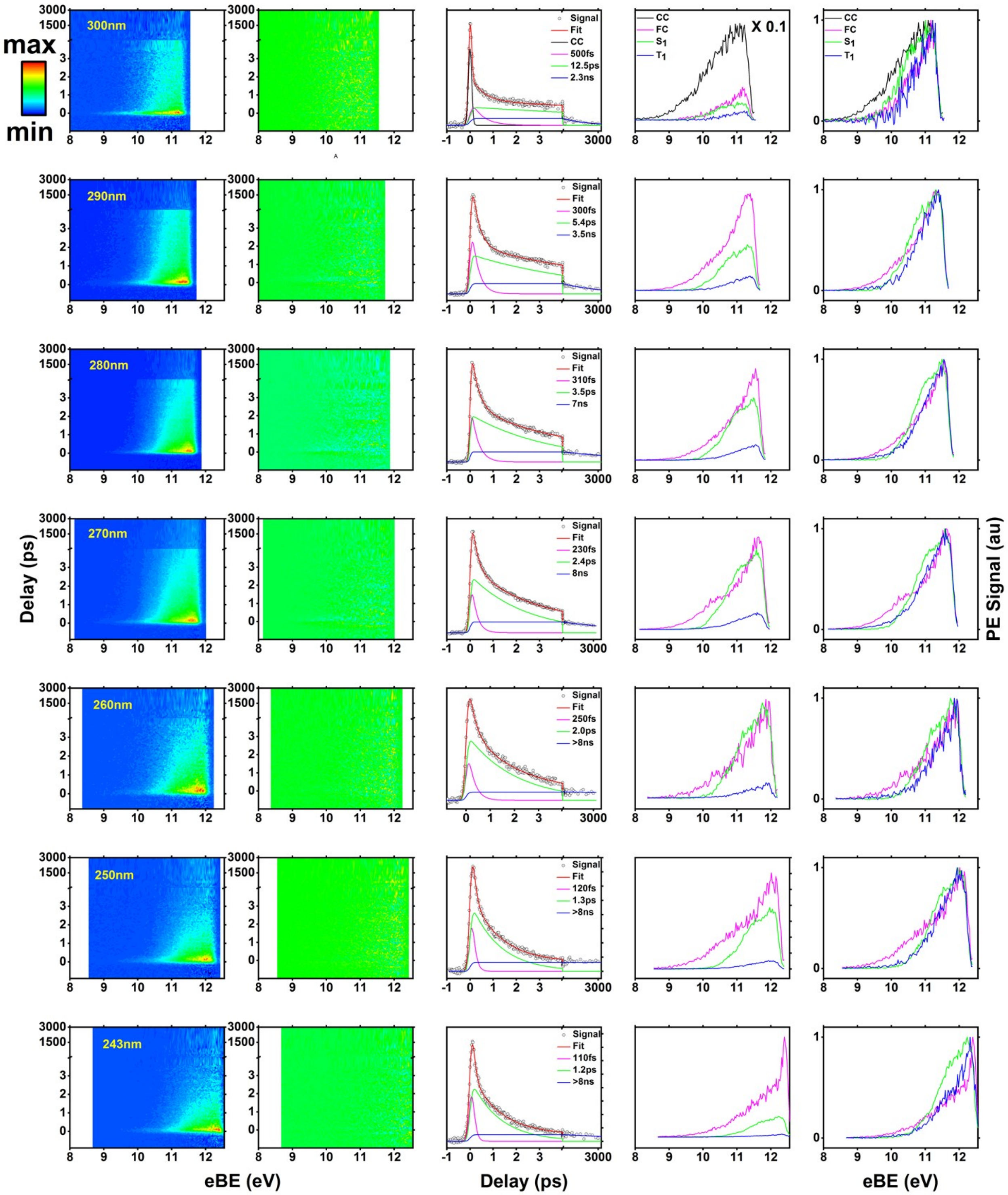

Figure S10 TRPES analysis of 2TC using a three-exponential parallel decay model. Each row corresponds to the TRPES data at a specific pump wavelength. From left to right, the columns show: the TRPES signal, the residuals of the global fit, the energy-integrated time traces, the decay associated spectra (DAS), and the normalized DAS. The DAS of the ultrafast time constant at 300nm is reduced by a factor of 0.1.

Figures S9 and S10 show the TRPES analyses of 2TC using three-exponential sequential and parallel decay models, respectively. The two different fit models give similar values of time constants, but as expected the fit components show slightly different trends in relative amplitudes as the pump wavelength is changed (third and fourth columns of Figures S9 and S10). The TRPES analysis of 2TC with a three-exponential sequential model is chosen over the parallel model as it agrees best with theoretical, multi-step decay dynamics of thiol 2TC.

S4. Fit equation

All TRPES data (pump wavelengths: 290nm, 280nm, 270nm, 260nm, 250nm, and 243nm; all with two-photon 330nm probe) were analyzed with a three-exponential sequential decay model. For the 300nm data, an additional Gaussian function was added to the fit function to account for a spike within the cross-correlation that may be due to a non-negligible probe-pump contribution. The analysis is illustrated in Figure S9. For comparison, the data are also analyzed with a parallel decay model which is shown in Figure S10.

The three-step sequential exponential decay function corresponds to the decay processes: A → B → C→D.

$$
\begin{aligned}
x(t,E) &= x(0,E) + \sigma_A(E) e^{-k_1 t} e^{\left(\frac{IRF * k_1}{4\sqrt{ln2}}\right)^2} * \frac{1}{2}\left[1 + \operatorname{erf}\left\{\left(\frac{2\sqrt{ln2} * t}{IRF}\right) - \left(\frac{IRF * k_1}{4\sqrt{ln2}}\right)\right\}\right] + \sigma_B(E) * \\
&* \frac{1}{2}\left[1 + \operatorname{erf}\left\{\left(\frac{2\sqrt{ln2} * t}{IRF}\right) - \left(\frac{IRF * k_1}{4\sqrt{ln2}}\right)\right\}\right] + \sigma_B(E) * \frac{k_1}{k_1 - k_2} * e^{-k_2 t} e^{\left(\frac{IRF * k_2}{4\sqrt{ln2}}\right)^2} * \frac{1}{2} \\
&\left[1 + \operatorname{erf}\left\{\left(\frac{2\sqrt{ln2} * t}{IRF}\right) - \left(\frac{IRF * k_2}{4\sqrt{ln2}}\right)\right\}\right] + \sigma_C(E) * \frac{k_1 k_2}{(k_2 - k_1)(k_3 - k_1)} * e^{-k_1 t} e^{\left(\frac{IRF * k_1}{4\sqrt{ln2}}\right)^2} * \frac{1}{2} \\
&\left[1 + \operatorname{erf}\left\{\left(\frac{2\sqrt{ln2} * t}{IRF}\right) - \left(\frac{IRF * k_1}{4\sqrt{ln2}}\right)\right\}\right] + \sigma_C(E) * \frac{k_1 k_2}{(k_1 - k_2)(k_3 - k_2)} * e^{-k_2 t} e^{\left(\frac{IRF * k_2}{4\sqrt{ln2}}\right)^2} * \frac{1}{2} \\
&\left[1 + \operatorname{erf}\left\{\left(\frac{2\sqrt{ln2} * t}{IRF}\right) - \left(\frac{IRF * k_2}{4\sqrt{ln2}}\right)\right\}\right] + \sigma_C(E) * \frac{k_1 k_2}{(k_1 - k_3)(k_2 - k_3)} * e^{-k_3 t} e^{\left(\frac{IRF * k_3}{4\sqrt{ln2}}\right)^2} * \frac{1}{2} \\
&\left[1 + \operatorname{erf}\left\{\left(\frac{2\sqrt{ln2} * t}{IRF}\right) - \left(\frac{IRF * k_3}{4\sqrt{ln2}}\right)\right\}\right]
\end{aligned}
$$

In the fit equation, $\sigma_A(E)$, $\sigma_B(E)$, and $\sigma_C(E)$ are the evolution-associated spectra, and $k_1 = 1/\tau_1$, $k_2 = 1/\tau_2$, and $k_3 = 1/\tau_3$ are the rate constants of the consecutive decay processes. The instrument response function (IRF) is characterized by the FWHM of the Gaussian cross-correlation.

The fit equation with an additional Gaussian function to account for probe-pump contributions in the cross-correlation region is as follows:

$$
\begin{aligned}
x(t,E) &= x(0,E) + \sigma_{A'}(E)e^{-\left(\frac{x-x0}{\sqrt{2}w}\right)^2} + \sigma_A(E)e^{-k_1 t}e^{\left(\frac{IRF*k_1}{4\sqrt{ln2}}\right)^2} * \frac{1}{2}\left[1+\text{erf}\left\{\left(\frac{2\sqrt{ln2}*t}{IRF}\right)-\left(\frac{IRF}{4\sqrt{}}\right.\right.\right. \text{[illegible]} \\
&\frac{k_1}{k_2-k_1} * e^{-k_1 t}e^{\left(\frac{IRF*k_1}{4\sqrt{ln2}}\right)^2} * \frac{1}{2}\left[1+\text{erf}\left\{\left(\frac{2\sqrt{ln2}*t}{IRF}\right)-\left(\frac{IRF*k_1}{4\sqrt{ln2}}\right)\right\}\right] + \sigma_B(E) * \frac{k_1}{k_1-k_2} * \text{[illegible]} \\
&e^{\left(\frac{IRF*k_2}{4\sqrt{ln2}}\right)^2} * \frac{1}{2}\left[1+\text{erf}\left\{\left(\frac{2\sqrt{ln2}*t}{IRF}\right)-\left(\frac{IRF*k_2}{4\sqrt{ln2}}\right)\right\}\right] + \sigma_C(E) * \frac{k_1k_2}{(k_2-k_1)(k_3-k_1)} * e^{-k_1} \text{[illegible]} \\
&* \frac{1}{2}\left[1+\text{erf}\left\{\left(\frac{2\sqrt{ln2}*t}{IRF}\right)-\left(\frac{IRF*k_1}{4\sqrt{ln2}}\right)\right\}\right] + \sigma_C(E) * \frac{k_1k_2}{(k_1-k_2)(k_3-k_2)} * e^{-k_2 t}e^{\left(\frac{IRF*k_2}{4\sqrt{ln2}}\right)} \text{[illegible]} \\
&\left[1+\text{erf}\left\{\left(\frac{2\sqrt{ln2}*t}{IRF}\right)-\left(\frac{IRF*k_2}{4\sqrt{ln2}}\right)\right\}\right] + \sigma_C(E) * \frac{k_1k_2}{(k_1-k_3)(k_2-k_3)} * e^{-k_3 t}e^{\left(\frac{IRF*k_3}{4\sqrt{ln2}}\right)^2} * \text{[illegible]} \\
&\left[1+\text{erf}\left\{\left(\frac{2\sqrt{ln2}*t}{IRF}\right)-\left(\frac{IRF*k_3}{4\sqrt{ln2}}\right)\right\}\right]
\end{aligned}
$$

In the additional Gaussian function, $\sigma_{A'}(E)$, x0 and w are amplitude, time-zero, and FWHM respectively.

S5. Justification of fits

To justify the use of an exponential fit with three components, the inadequacy of two exponentials is demonstrated for the time trace at 290nm (Figure S11). The latter fit clearly indicates the requirement of a third component to describe the long-lived signal.

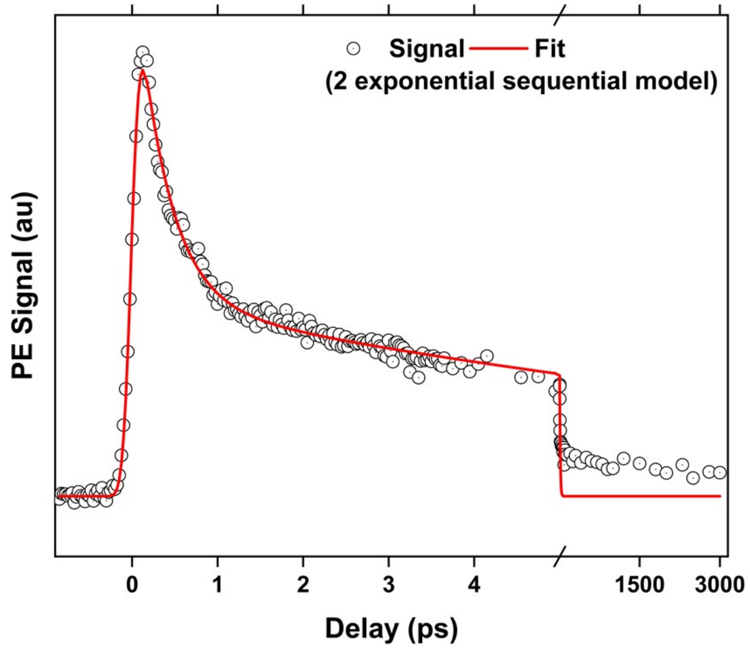

Figure S11 Fit of the energy integrated time trace at 290nm using a two-exponential sequential model.

The scree plot and the Singular Value Decomposition (SVD) vectors of the TRPES data at 290nm are shown in Figure S12. SVD analysis enables one to estimate the number of channels required to fit the data in global analysis. By increasing the number of independent linear singular vectors, it shows that the three vectors are significantly different from noise and adding a fourth channel does not improve the quality of the fit.

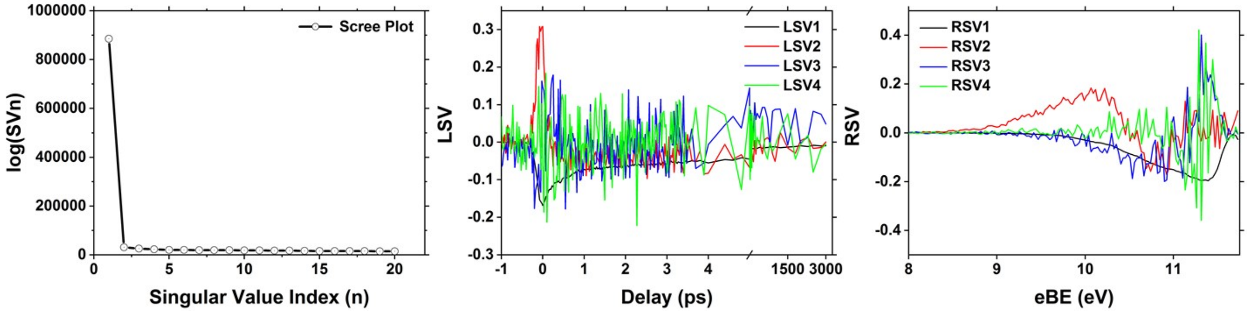

Figure S12: Scree plot, left singular values (LSV) plot, and right singular values (RSV) plot of TRPES data at 290nm.

The same conclusion is reached based on the residuals of the 2D global lifetime analysis of the TRPES data (Figure S9) which show a good quality of the fit with three exponentials.

S6. Cartesian coordinates of all optimized points at the XMS-CASPT2(10,8) ANO-R1 level of theory.

The following lists all 30 optimized structures in xyz format.

13
S_0 min a -717.785931856695
C +0.013215 +0.966035 +0.004083
C -1.246670 +0.328407 +0.006527
C -1.233324 -1.059115 +0.032807
C +1.025726 -1.073934 +0.021269
N +1.160059 +0.262642 +0.006477
N -0.102647 -1.801219 +0.045634
N +0.133610 +2.326352 -0.043147
S +2.562541 -1.937623 +0.016267
H +1.052682 +2.678676 +0.190946
H -0.644570 +2.875913 +0.290109
H -2.174170 +0.889911 -0.012905
H -2.167141 -1.616764 +0.045705
H +1.970192 -3.141822 +0.036997

13
S_0 min b -717.785411233123
C +0.006170 +0.984645 +0.003738
C -1.244948 +0.331924 +0.004840
C -1.214811 -1.055952 +0.032373
C +1.045185 -1.040696 +0.024327
N +1.164520 +0.297462 +0.008871
N -0.074884 -1.780319 +0.047531
N +0.108392 +2.347543 -0.044890
S +2.529062 -1.993397 +0.022478
H +1.020183 +2.711864 +0.199179
H -0.678105 +2.883853 +0.291204
H -2.178751 +0.882846 -0.015586
H -2.141518 -1.625385 +0.044577
H +3.321730 -0.910619 -0.005415

13
S_1 min nπ* -717.634174223328
C -0.015652 +0.905150 +0.021075
C -1.357579 +0.401348 +0.043954
C -1.409998 -1.003002 -0.102215
C +0.982118 -1.287638 +0.071261
N +1.034346 +0.106873 -0.041113
N -0.308988 -1.719605 -0.170881
N +0.215003 +2.259673 +0.005342
S +2.336904 -2.269517 -0.493925
H +1.149800 +2.534256 +0.280639
H -0.519013 +2.826448 +0.407375
H -2.235291 +1.034431 +0.069272
H -2.352058 -1.543922 -0.176432
H +2.829909 -2.547037 +0.726415

13
S_1 min ππ* a -717.627578800751
C -0.008579 +0.999353 -0.091142
C -1.282046 +0.357436 +0.046198
C -1.309107 -1.054134 -0.460771
C +0.949360 -1.022949 -0.145175
N +1.154235 +0.289878 -0.325975
N -0.203142 -1.737421 +0.002646
N +0.147934 +2.311438 +0.092183
S +2.433711 -1.949400 -0.080281
H +1.084227 +2.689200 +0.077676
H -0.649055 +2.920374 +0.183318
H -1.797212 +0.546566 +1.000452
H -2.241603 -1.607246 -0.526006
H +1.795348 -3.124987 +0.037804

13
S_1 min ππ* b -717.628619974160
C -0.018266 +1.012143 -0.088091
C -1.264821 +0.337696 +0.094015
C -1.283059 -1.062411 -0.421747
C +0.984537 -0.996866 -0.100831
N +1.162537 +0.327109 -0.314753
N -0.154917 -1.729693 -0.032232
N +0.127184 +2.321962 +0.115640
S +2.418764 -1.994414 -0.024070
H +1.062254 +2.706562 +0.087989
H -0.673253 +2.936070 +0.151121
H -1.890090 +0.603850 +0.955395
H -2.207331 -1.623730 -0.516852
H +3.259556 -0.963873 -0.215315

13
T_1 min ππ* a -717.654769254897
C +0.048005 +1.008316 +0.059483
C -1.241138 +0.344503 -0.230262
C -1.261875 -1.003374 +0.392969
C +0.969618 -1.056959 -0.046670
N +1.160926 +0.306321 +0.053295
N -0.159617 -1.750243 +0.240437
N +0.084733 +2.334844 +0.267145
S +2.443591 -1.944986 -0.358548
H +0.959025 +2.757078 +0.546996
H -0.773452 +2.846409 +0.387884
H -1.575475 +0.435905 -1.273118
H -2.146919 -1.440519 +0.843058
H +1.842081 -3.139837 -0.241900

13
T_1 min ππ* b -717.654206143875
C +0.043279 +1.030425 +0.076306
C -1.230696 +0.359249 -0.259355
C -1.260182 -0.994615 +0.351201
C +0.986464 -1.023109 -0.021092
N +1.165065 +0.341440 +0.094674
N -0.146951 -1.725270 +0.231709
N +0.058333 +2.355645 +0.301631
S +2.409170 -2.005793 -0.284928
H +0.914580 +2.779776 +0.630250
H -0.810975 +2.849916 +0.416819
H -1.535616 +0.457059 -1.310482
H -2.157105 -1.445654 +0.762483
H +3.226859 -0.945299 -0.375991

13
T_1 min nπ* -717.644530969145
C -0.002461 +0.886716 -0.021050
C -1.351148 +0.393345 -0.024616
C -1.430101 -1.001346 +0.095425
C +0.931057 -1.328368 -0.124607
N +1.029050 +0.073005 -0.021812
N -0.357075 -1.759480 +0.180371
N +0.237919 +2.239646 -0.014136
S +2.259488 -2.327747 +0.485304
H +1.173057 +2.502845 -0.301427
H -0.493855 +2.812978 -0.412213
H -2.218397 +1.042618 -0.022359
H -2.390633 -1.504768 +0.201100
H +2.962602 -2.331987 -0.660748

13
S_1T_2 MECP a -717.6327144519
C -0.002962 +0.915455 -0.085873
C -1.304625 +0.286516 -0.127966
C -1.257647 -1.119281 +0.087534
C +1.117854 -1.165543 -0.090396
N +1.119098 +0.232007 -0.006604
N -0.118471 -1.756503 +0.181057
N +0.074701 +2.276669 -0.073545
S +2.602212 -1.944750 +0.460023
H +0.983194 +2.670577 -0.279819
H -0.719558 +2.784463 -0.434024
H -2.229158 +0.849884 -0.156104
H -2.169865 -1.708749 +0.170831
H +2.336891 -3.113634 -0.148258

13
S_1T_2 MECP b -717.6326696963
C -0.027051 +0.963113 -0.030268
C -1.308148 +0.298948 -0.041070
C -1.213317 -1.120404 +0.026576
C +1.132946 -1.048159 +0.420322
N +1.116809 +0.318116 +0.097820
N -0.053478 -1.715492 +0.153751
N +0.015688 +2.315611 -0.165373
S +2.604851 -1.988011 +0.162182
H +0.922381 +2.732145 -0.321921
H -0.776057 +2.772333 -0.590606
H -2.248951 +0.833125 -0.081654
H -2.098023 -1.752898 -0.024996
H +3.384395 -1.091043 +0.788516

13
T_2T_1 MECI a -717.638042320831
C +0.030303 +0.949783 +0.069716
C -1.298304 +0.371600 +0.090011
C -1.318803 -1.047801 -0.011366
C +1.034539 -1.148449 -0.361728
N +1.109914 +0.230432 -0.099688
N -0.228823 -1.742671 -0.185866
N +0.164753 +2.291881 +0.302127
S +2.461563 -2.054777 +0.153231
H +1.065008 +2.683127 +0.055619
H -0.631366 +2.870419 +0.074867
H -2.191977 +0.961547 +0.254083
H -2.263514 -1.590022 +0.015890
H +2.140773 -3.156958 -0.545963

13
T_2T_1 MECI b -717.637062178310
C +0.017727 +0.989685 +0.085560
C -1.286848 +0.368302 +0.132916
C -1.268479 -1.048627 -0.019135
C +1.097060 -1.089242 -0.294649
N +1.123909 +0.309964 -0.075441
N -0.154894 -1.696183 -0.216247
N +0.104968 +2.342248 +0.299318
S +2.423793 -2.073152 +0.338286
H +0.988413 +2.757228 +0.031477
H -0.708832 +2.883949 +0.043533
H -2.199576 +0.930323 +0.288730
H -2.197430 -1.616230 -0.057353
H +3.364889 -1.203818 -0.067340

13
S_1S_0 MECI C_2 puck -717.609919198276
C -0.059058 +0.878904 -0.001147
C -1.322611 +0.252695 -0.267285
C -1.287415 -1.059228 +0.170845
C +1.039827 -1.155556 -0.044296
N +0.969505 +0.165973 +0.523247
N -0.141892 -1.551778 +0.694875
N +0.143588 +2.186836 -0.188900
S +2.587334 -1.954150 +0.273717
H +1.056303 +2.573753 -0.000108
H -0.589043 +2.782800 -0.537640
H -2.184203 +0.754718 -0.687888
H -2.154488 -1.720125 +0.167220
H +2.373819 -2.947729 -0.605784

13
S_1S_0 MECI oop-NH_2 a -717.609559332477
C -0.042268 +0.988707 -0.490551
C -1.321693 +0.323968 -0.102782
C -1.289835 -1.012003 +0.104169
C +0.998001 -1.098976 -0.191671
N +0.808674 -0.070976 -1.117901
N -0.056998 -1.732254 +0.211137
N +0.613283 +1.641311 +0.600403
S +2.643645 -1.577329 +0.123005
H +1.130532 +2.461681 +0.294568
H -0.047224 +1.915198 +1.322099
H -2.205150 +0.930393 +0.078508
H -2.164469 -1.601454 +0.359180
H +2.267178 -2.498965 +1.026648

13
S_1S_0 MECI oop-NH_2 b -717.610103526308
C -0.055596 +1.003658 -0.476875
C -1.330544 +0.323267 -0.120612
C -1.273677 -1.010133 +0.120778
C +0.996774 -1.056705 -0.183584
N +0.799080 -0.044119 -1.134992
N -0.027814 -1.693981 +0.275272
N +0.618984 +1.658507 +0.593900
S +2.625011 -1.486174 +0.256892
H +1.110048 +2.492859 +0.284353
H -0.014546 +1.902523 +1.349097
H -2.237917 +0.902685 +0.025916
H -2.147065 -1.610896 +0.352972
H +3.171616 -0.753992 -0.730302

13
S_1S_0 MECI oop-SH a -717.629263634532
C +0.195734 +0.882378 -0.132250
C -0.920915 +0.087519 +0.445704
C -0.999984 -1.221038 +0.042284
C +1.216402 -1.099809 -0.631862
N +1.202040 +0.360780 -0.758864
N -0.123272 -1.612208 -0.936938
N +0.075397 +2.222602 -0.012410
S +1.936555 -1.669109 +0.921236
H +0.849713 +2.796027 -0.317056
H -0.626326 +2.627701 +0.583842
H -1.712637 +0.563342 +1.015295
H -1.855061 -1.853890 +0.270546
H +2.101538 -2.934657 +0.496727

13
S_1S_0 MECI oop-SH b -717.626989253254
C +0.211226 +0.927843 -0.179472
C -0.823775 +0.105413 +0.506506
C -0.898118 -1.200118 +0.111483
C +1.251684 -1.011651 -0.718184
N +1.213540 +0.424931 -0.842937
N -0.085880 -1.549599 -0.953406
N +0.058668 +2.263748 -0.082758
S +2.166688 -1.725261 +0.641918
H +0.764312 +2.858656 -0.493396
H -0.644925 +2.668798 +0.510493
H -1.571069 +0.559503 +1.149755
H -1.685656 -1.880114 +0.428446
H +1.885311 -0.822424 +1.606865

13
S_0S_1 MECI C_5 a -717.641684323069
C -0.000616 +1.018320 +0.137036
C -1.223179 +0.291406 +0.551761
C -1.300741 -0.830967 -0.426726
C +0.893626 -1.026489 +0.019351
N +1.142061 +0.311234 +0.079154
N -0.245302 -1.638954 -0.475534
N +0.000380 +2.311948 -0.185617
S +2.280872 -2.035144 +0.317325
H +0.843579 +2.748914 -0.530694
H -0.849413 +2.843180 -0.085091
H -0.952387 -0.180027 +1.518457
H -2.146015 -1.035678 -1.081649
H +1.631204 -3.159632 -0.026846

13
S_0S_1 MECI C_5 b -717.641276240673
C -0.002004 +1.036720 +0.142175
C -1.207008 +0.291349 +0.569090
C -1.282364 -0.823033 -0.421449
C +0.920621 -0.990574 +0.004999
N +1.154586 +0.349656 +0.077940
N -0.214021 -1.608750 -0.491661
N -0.029617 +2.327172 -0.195922
S +2.255151 -2.081858 +0.249194
H +0.795163 +2.772707 -0.572596
H -0.889452 +2.842025 -0.095066
H -0.924765 -0.184618 +1.529686
H -2.135621 -1.038239 -1.062184
H +3.101203 -1.100064 +0.603599

13
S_0S_1 MECI C_6 a -717.616358541970
C -0.002805 +0.998318 -0.062921
C -1.234558 +0.309394 -0.005221
C -1.228498 -1.086478 -0.556717
C +0.981534 -0.937883 -0.045273
N +1.179316 +0.329032 -0.343072
N -0.217089 -1.511699 +0.359619
N +0.122435 +2.313652 +0.174675
S +2.353586 -2.020054 -0.150522
H +1.047300 +2.716184 +0.164358
H -0.683181 +2.909253 +0.269639
H -1.981476 +0.607873 +0.732788
H -2.150719 -1.662632 -0.551694
H +1.653789 -3.088968 +0.266125

13
S_0S_1 MECI C_6 b -717.615246477184
C +0.000925 +1.015117 -0.073036
C -1.209569 +0.301804 +0.017633
C -1.192594 -1.096412 -0.536143
C +1.000903 -0.920164 -0.023081
N +1.185241 +0.351041 -0.345198
N -0.193160 -1.502292 +0.371607
N +0.108243 +2.334962 +0.154041
S +2.333507 -2.046029 -0.034758
H +1.026837 +2.750961 +0.145974
H -0.706993 +2.919642 +0.232363
H -1.939550 +0.568031 +0.782931
H -2.105783 -1.686723 -0.561495
H +3.215088 -1.115531 -0.440571

13
S_0T_1 MECP C_2 puck -717.6228303538
C -0.070624 +0.876022 -0.003993
C -1.381887 +0.306562 -0.138541
C -1.331406 -1.043087 +0.149087
C +1.056344 -1.232145 -0.099271
N +0.966328 +0.126372 +0.390494
N -0.196114 -1.623728 +0.546905
N +0.157159 +2.174396 -0.231620
S +2.522826 -2.052975 +0.505204
H +1.098823 +2.530591 -0.167583
H -0.592120 +2.792569 -0.494360
H -2.259276 +0.848402 -0.468359
H -2.212596 -1.684394 +0.100874
H +2.674209 -2.811475 -0.591978

13
S_0T_1 MECP oop-NH_2 a -717.6129045721
C -0.015091 +0.972877 -0.434706
C -1.289542 +0.319572 -0.094631
C -1.287521 -1.038674 +0.061158
C +0.994426 -1.160255 -0.199107
N +0.786163 -0.079854 -1.060822
N -0.080258 -1.791418 +0.147811
N +0.667783 +1.751814 +0.543616
S +2.634813 -1.618595 +0.164713
H +0.909123 +2.674630 +0.194784
H +0.131774 +1.850705 +1.400576
H -2.166399 +0.919481 +0.134051
H -2.183789 -1.606583 +0.286545
H +2.232195 -2.524399 +1.072825

13
S_0T_1 MECP oop-NH_2 b -717.6134688521
C -0.028806 +0.990235 -0.415051
C -1.291710 +0.321116 -0.088218
C -1.270697 -1.042005 +0.057524
C +1.004348 -1.122644 -0.185805
N +0.776344 -0.048677 -1.050663
N -0.054653 -1.772766 +0.170418
N +0.644493 +1.802713 +0.537658
S +2.618912 -1.551901 +0.296534
H +0.852499 +2.728301 +0.175256
H +0.124961 +1.893285 +1.405649
H -2.180575 +0.904993 +0.135474
H -2.163301 -1.621662 +0.267358
H +3.202538 -0.853491 -0.693319

13
S_0T_1 MECP oop-SH a -717.6312279229
C +0.139648 +0.885314 -0.136055
C -0.992980 +0.124604 +0.411694
C -1.029762 -1.194843 +0.044261
C +1.207281 -1.126044 -0.619535
N +1.182825 +0.329550 -0.685579
N -0.110617 -1.570026 -0.911552
N +0.062562 +2.237455 -0.063025
S +1.990364 -1.707293 +0.901658
H +0.906261 +2.754855 -0.269292
H -0.605429 +2.661195 +0.559862
H -1.780034 +0.593467 +0.991646
H -1.825219 -1.883026 +0.316405
H +2.194285 -2.955570 +0.445766

13
S_0T_1 MECP oop-SH b -717.6285514037
C +0.165111 +0.933806 -0.174699
C -0.880577 +0.136327 +0.482954
C -0.900923 -1.172758 +0.111188
C +1.263367 -1.029739 -0.725174
N +1.179569 +0.410017 -0.819069
N -0.049521 -1.508433 -0.924527
N +0.074006 +2.275283 -0.071140
S +2.264508 -1.706042 +0.590442
H +0.812355 +2.842967 -0.458062
H -0.682046 +2.711906 +0.426241
H -1.597083 +0.570148 +1.171488
H -1.614692 -1.908837 +0.472556
H +1.807930 -0.934922 +1.603115

13
S_0T_1 MECP C_5 a -717.6473239169
C +0.023039 +1.035472 +0.004532
C -1.176078 +0.287523 -0.431289
C -1.260917 -0.905715 +0.440473
C +0.921666 -1.027162 -0.097014
N +1.141392 +0.328694 -0.018369
N -0.177998 -1.694154 +0.354769
N -0.006172 +2.306212 +0.430146
S +2.342189 -1.952334 -0.537265
H +0.827324 +2.718356 +0.823109
H -0.851779 +2.846201 +0.364647
H -1.097555 +0.019184 -1.497055
H -2.067325 -1.135050 +1.129827
H +1.731715 -3.129769 -0.325742

13
S_0T_1 MECP C_5 b -717.6454560550
C +0.011240 +1.054542 +0.061941
C -1.157923 +0.312469 -0.454943
C -1.284093 -0.910034 +0.358584
C +0.926985 -1.014177 -0.033797
N +1.132527 +0.345483 +0.096619
N -0.195788 -1.700235 +0.273497
N -0.026974 +2.330987 +0.462452
S +2.323368 -2.003459 -0.404487
H +0.791336 +2.743358 +0.886496
H -0.879207 +2.860530 +0.393544
H -1.028747 +0.095807 -1.527411
H -2.087733 -1.135249 +1.052352
H +3.137235 -0.946251 -0.551619

13
S_0T_1 MECP C_6 a -717.6294241815
C +0.008457 +0.981627 -0.010042
C -1.213346 +0.289266 -0.054275
C -1.199748 -1.120140 -0.473633
C +1.021234 -0.944951 -0.009532
N +1.221132 +0.330844 -0.229830
N -0.157700 -1.489670 +0.474907
N +0.106984 +2.298811 +0.212953
S +2.326390 -2.059070 -0.330833
H +1.024891 +2.716299 +0.224171
H -0.709328 +2.882752 +0.290464
H -2.113192 +0.773660 +0.324278
H -2.129869 -1.670893 -0.328067
H +1.653728 -3.112543 +0.161224

13
S_0T_1 MECP C_6 b -717.6284850029
C +0.013442 +0.993745 +0.005175
C -1.194580 +0.282544 -0.066856
C -1.145473 -1.130339 -0.469415
C +1.059170 -0.915646 +0.053905
N +1.244476 +0.361489 -0.177402
N -0.127283 -1.467297 +0.515497
N +0.086022 +2.314648 +0.219344
S +2.334525 -2.084416 -0.167908
H +0.996338 +2.746264 +0.258133
H -0.741084 +2.886006 +0.272257
H -2.113028 +0.757604 +0.277142
H -2.069023 -1.697461 -0.349646
H +3.179594 -1.172736 -0.679960
